# The *Staphylococcus aureus* superantigen SElX is a bifunctional toxin that inhibits neutrophil function

**DOI:** 10.1371/journal.ppat.1006461

**Published:** 2017-09-07

**Authors:** Stephen W. Tuffs, David B. A. James, Jovanka Bestebroer, Amy C. Richards, Mariya I. Goncheva, Marie O’Shea, Bryan A. Wee, Keun Seok Seo, Patrick M. Schlievert, Andreas Lengeling, Jos A. van Strijp, Victor J. Torres, J. Ross Fitzgerald

**Affiliations:** 1 The Roslin Institute, University of Edinburgh, Easter Bush Campus, Midlothian, Scotland, United States of America; 2 Department of Microbiology, New York University School of Medicine, New York, NY, United Kingdom; 3 Department Medical Microbiology, UMC Utrecht, Heidelberglaan 100, 3584 CX Utrecht, The Netherlands; 4 Department of Basic Sciences, College of Veterinary Medicine, Mississippi State University, Starkville, MS, United States; 5 Department of Microbiology, University of Iowa, Carver College of Medicine, Iowa City, Iowa, United States of America; University of Tubingen, GERMANY

## Abstract

Bacterial superantigens (SAgs) cause Vβ-dependent T-cell proliferation leading to immune dysregulation associated with the pathogenesis of life-threatening infections such as toxic shock syndrome, and necrotizing pneumonia. Previously, we demonstrated that staphylococcal enterotoxin-like toxin X (SElX) from *Staphylococcus aureus* is a classical superantigen that exhibits T-cell activation in a Vβ-specific manner, and contributes to the pathogenesis of necrotizing pneumonia. Here, we discovered that SElX can also bind to neutrophils from human and other mammalian species and disrupt IgG-mediated phagocytosis. Site-directed mutagenesis of the conserved sialic acid-binding motif of SElX abolished neutrophil binding and phagocytic killing, and revealed multiple glycosylated neutrophil receptors for SElX binding. Furthermore, the neutrophil binding-deficient mutant of SElX retained its capacity for T-cell activation demonstrating that SElX exhibits mechanistically independent activities on distinct cell populations associated with acquired and innate immunity, respectively. Finally, we demonstrated that the neutrophil-binding activity rather than superantigenicity is responsible for the SElX-dependent virulence observed in a necrotizing pneumonia rabbit model of infection. Taken together, we report the first example of a SAg, that can manipulate both the innate and adaptive arms of the human immune system during *S*. *aureus* pathogenesis.

## Author summary

*Staphylococcus aureus* is a bacterial pathogen responsible for an array of disease types in healthcare and community settings. One of the keys to the success of this pathogen is its ability to subvert the immune system of the host. Here we demonstrate that the superantigen (SAg) staphylococcal enterotoxin-like toxin X (SElX) contributes to immune evasion by inducing unregulated T-cell proliferation, and by inhibition of phagocytosis by neutrophils. We observed that the capacity to bind neutrophils appears to be central to the SElX-dependent toxicity observed in a necrotising pneumonia infection model in rabbits. We report the first example of a staphylococcal SAg with two independent immunomodulatory functions acting on distinct immune cell types.

## Introduction

*Staphylococcus aureus* is an opportunistic pathogen responsible for a wide array of human diseases in both the hospital and community settings [1]. The diversity of disease types and the strain-dependent variation in pathogenic potential is due in part to the large array of virulence factors that are produced by *S*. *aureus* [1]. The staphylococcal superantigens (SAgs) are a family of at least 26 secreted proteins that modulate the immune system by stimulating dysregulated T-cell proliferation [2–4], contributing to a variety of different diseases including toxic shock syndrome, necrotizing pneumonia and Kawasaki disease [2]. The diversity of SAgs produced by *S*. *aureus* strains facilitates interaction with the large repertoire of variable-β chains (Vβ) found in the T-cell receptor leading to dysregulation of a critical component of the adaptive immune response [2, 5].

The SAg SElX is encoded in the core genome of over 95% of *S*. *aureus* isolates and contributes to lethality in a rabbit model of necrotising pneumonia [6]. Although a member of the SAg family, SElX exhibits greater sequence homology with the staphylococcal superantigen-like protein (SSl) family comprising of proteins that are structurally similar to SAgs but lack the capacity to induce Vβ-specific T-cell proliferation [5]. The SSls are associated with a diversity of immune evasion functions including the blockade of complement activation, interference with bacterial cell wall opsonisation (e.g. SSl7 and SSl10) and disruption of neutrophil function (e.g. SSl3, SSl4 and SSl5) [7–11]. Of note, SSl5 can bind to neutrophils via a direct interaction with CD162 (P-selectin glycoprotein ligand-1; PSGL-1), reducing neutrophil migration [7, 12], and SSl3 and SSl4 are toll-like receptor 2 antagonists which prevent neutrophil activation by bacterial lipopeptides [11, 13, 14]. Fevre et al. (2014) previously demonstrated that SElX can interact with neutrophils and monocytes, binding via the CD162 molecule on the surface of neutrophils [15]. However, very high concentrations of SElX were required for a relatively low-affinity interaction suggesting that CD162 may not be the main neutrophil receptor involved [15].

In addition to SSls, *S*. *aureus* produces other molecules which subvert the innate immune response including chemotaxis inhibitory protein of staphylococcus (CHIPS), which binds to the formylated peptide and C5a receptors on neutrophils, blocking chemotaxis to the site of infection [16]. Furthermore, the formyl peptide receptor-like 1 inhibitor (FLIPr) and its homologue FLIPr-like can antagonise the formylated peptide receptor and bind to Fcγ receptors disrupting IgG-mediated phagocytosis of neutrophils [17, 18]. Other examples of multifunctional determinants include; extracellular adherence protein (EAP) which can act as a host cell invasin and inhibit the activity of neutrophil elastase, Panton–Valentine leucocidin (PVL) which has been demonstrated to induce inflammation independent of cell lysis, and collagen binding protein (CNA) which, in addition to its role binding to extracellular collagen, can bind C1q and block the complement cascade [19–21]. These examples of multi-functionality highlight the apparent functional redundancy exhibited by *S*. *aureus* with regard to pathogenesis, providing a robust, multi-faceted response to innate immunity during the early stages of infection.

In the current study, we further investigated the role of SElX in *S*. *aureus* disease pathogenesis. We discovered that SElX binds to neutrophils via multiple glycosylated neutrophil surface receptors, inhibiting phagocytosis and contributing to the pathogenesis of severe lung infection. Importantly, the neutrophil binding and superantigenic functions of SElX are mechanistically independent providing a bi-functional disruption of both the innate and adaptive arms of the human immune system.

## Results

### SElX binds to neutrophils and monocytes from multiple mammalian species

**Fig 1:**
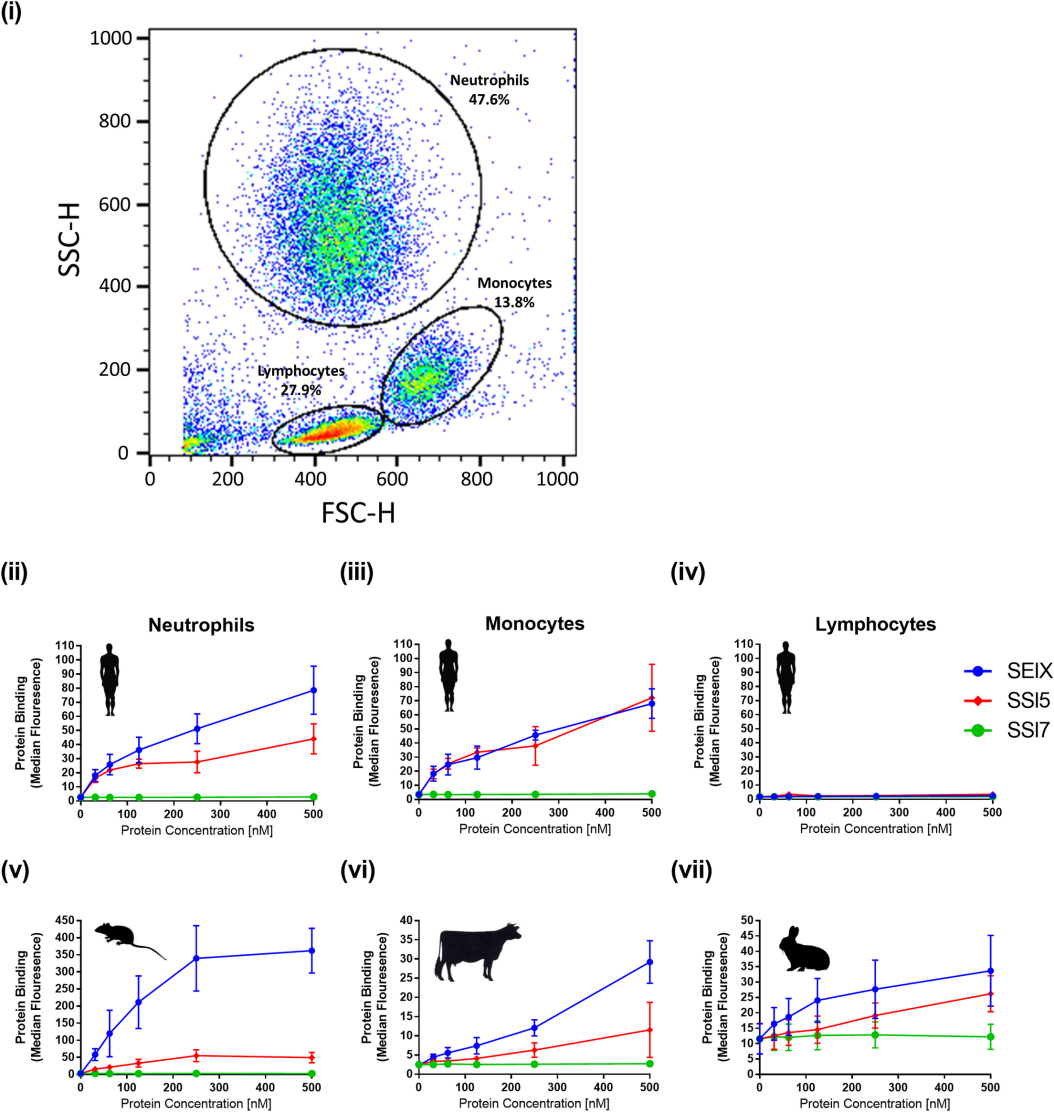
SElX binds to human monocytes and neutrophils from multiple mammalian species. Flow cytometry analysis of recombinant staphylococcal proteins binding to isolated human cells. Cell type was determined by forward (FSC-H) and sideways (SSC-H) scatter (i). SElX binding to human neutrophils (ii) monocytes (iii) and lymphocytes (iv) in addition to neutrophils isolated from mice (v), cattle (vi) and rabbits (vii), was examined. For all graphs binding was detected using mouse anti-HIS-FITC IgG binding to the 6 x HIS-tag on the recombinant proteins. Mean median fluorescence of three donors is shown ± standard error of the mean (SEM). SSl5 and SSl7 were used as positive and negative controls, respectively. The same legend is used for all graphs.

In order to test the hypothesis that SElX can bind to human leukocytes, recombinant SElX was incubated with human leukocytes isolated from healthy donors. SElX bound to both human neutrophils and monocytes at a high level, comparable to or greater than the known neutrophil-binding protein SSl5 (Fig 1ii and 1iii). In addition, SElX exhibited low level binding to CD4^+^ and CD8^+^ T-lymphocyte subsets but not to B-lymphocytes (CD19) (S1 Fig).

In order to examine the host-dependent binding of SElX, neutrophils from cattle, rabbits and mice were employed. SElX exhibited a higher level of binding to murine neutrophils compared to human neutrophils, but this was reduced compared to bovine and laprine neutrophils. However, binding to neutrophils from all species was observed at a concentration below 100 nM (Fig 1v, 1vi and 1vii). Overall SElX exhibited binding with neutrophils from multiple mammalian species at a level that exceeded that of the known neutrophil-binding protein SSl5.

### SElX binding to neutrophils is dependent on a conserved sialic acid-binding motif

**Fig 2:**
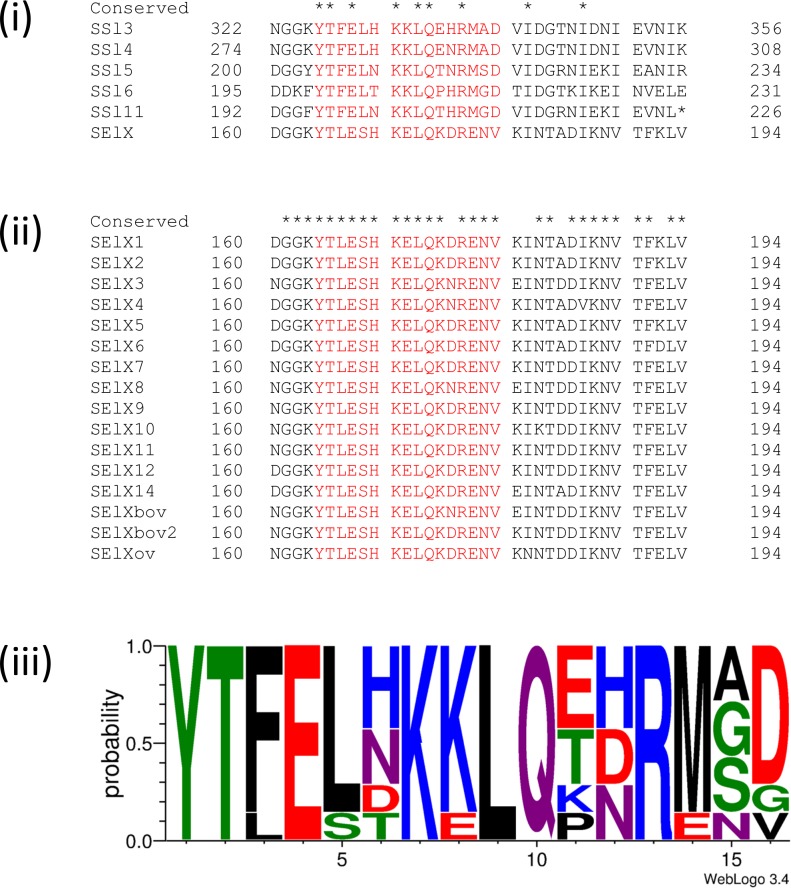
SElX protein sequence encodes a conserved sialic acid-binding motif. (i) A conserved glycan binding motif (colored red) is present in the amino acid sequences of characterised SSl-proteins and SElX. (ii) The sialic acid-binding motif is conserved in all 17 alleles of SElX. (iii) Amino acid conservation across the sialic acid-binding region of 7 staphylococcal neutrophil binding proteins (SSl2, SSl3, SSl4 SSl5, SSl6, SSl11 and SElX). The probability of residues at each position is proportional to the size of the letters. Image generated using the weblogo 3.4 program (http://weblogo.threeplusone.com/). Chemical property color scheme: green is polar, blue is basic, red is acidic, purple is neutral and black is hydrophobic. Sequences were obtained from previously published work [6, 23].

Amino acid sequence alignment of SElX with previously characterised SSl-proteins revealed a conserved sialic acid-binding motif (YTxExxKxLQx[H/N/D]Rxx[D/E]) matching 54% amino acid sequence identity with that of SSl5 (Fig 2) [7, 11, 22, 23]. Of note, 4 amino acids of the motif have been demonstrated to interact with sialic-Lewis X (sLEX) [23], and 3 of these residues (E154, K156, and Q169) are conserved in SElX (Fig 2) and the 5 SSls that contain the motif (Fig 2). The fourth residue ([H/N/D]161) identified to interact with sLEX in crystallography studies [23], varies among the SSl-proteins and allelic variants of SElX encoded by different strains suggesting that this residue is not essential for sialic acid binding (Fig 2). SElX exhibits considerable allelic variation, with at least 17 allelic variants, and a total of 40 (13%) variable amino acid positions identified [6]. However the sialic acid binding motif contains only one variable residue among all known allelic variants, consistent with functionality (Fig 2).

**Fig 3:**
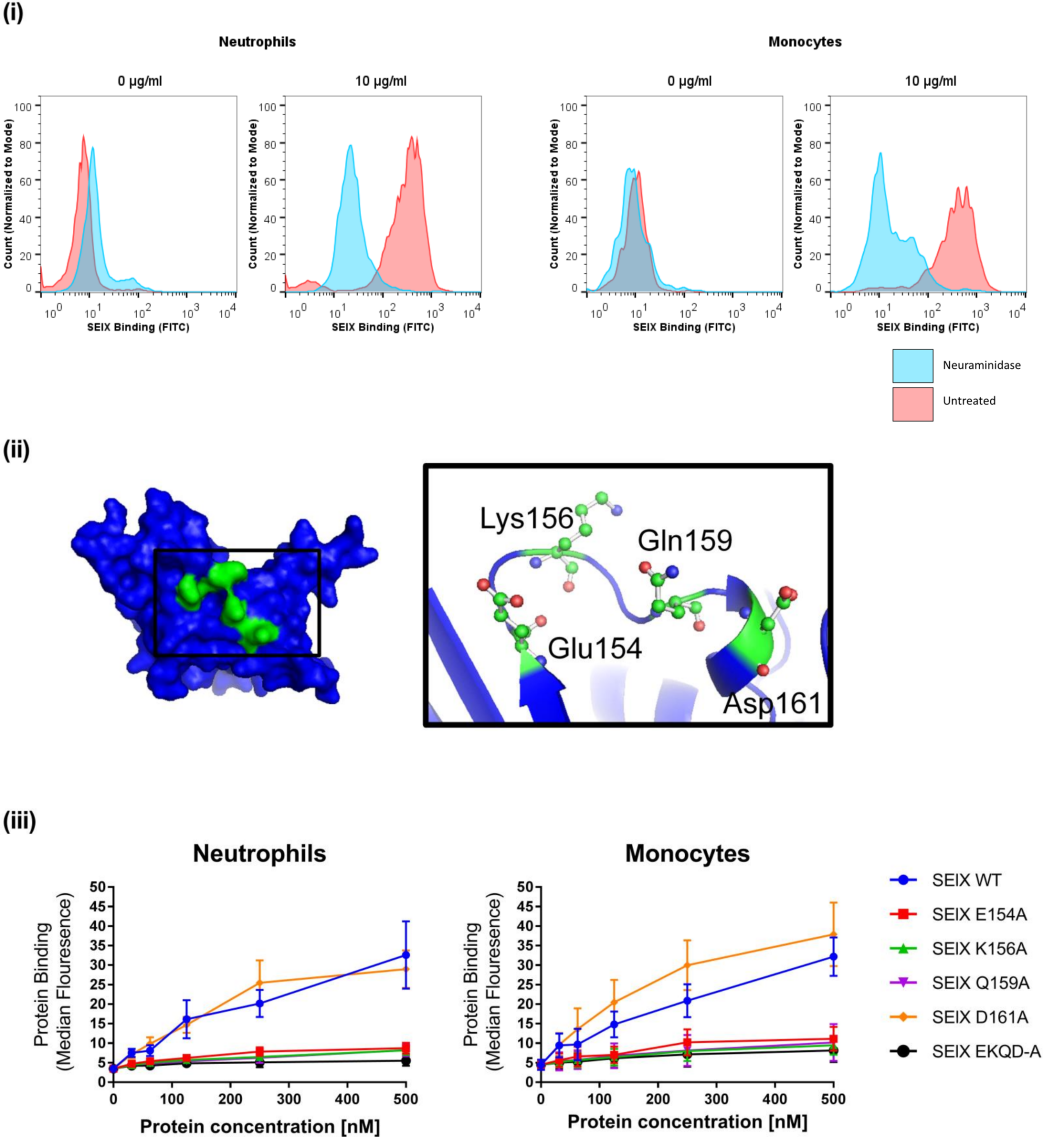
SElX interaction with neutrophils is dependent upon the sialic acid binding motif. (i) Flow cytometry analysis of SElX binding to human neutrophils and monocytes pretreated with 0.2 U/ml neuraminidase versus unteated cells. 6 x HIS-tagged SElX binding was detected using mouse anti-His FITC-labelled IgG. (ii) Hypothetical protein model of SElX indicating the location of predicted sialic acid binding residues (colored green). The motif residues are magnified in the black box to indicate their predicted atomic structure in the binding pocket. (iii) Flow cytometry analysis of site-directed alanine-replacement mutants of SElX binding to human neutrophils. 6 x HIS-tagged SElX binding was detected using Mouse anti-HIS FITC-labelled IgG.

To examine the hypothesis that SElX binding is sialic acid-dependent, neutrophils and monocytes were pre-treated with neuraminidase prior to binding. Neuraminidase treatment abolished the binding of SElX to both neutrophils and macrophages, suggesting that the interaction is sialic acid-dependent (Fig 3i). This is consistent with the observation of Fevre et al (2014) who also noted a sialic acid requirement for SElX binding [15]. In order to investigate the role of the predicted sialic acid-binding motif of SElX in neutrophil binding, single site-directed mutants of each of the four predicted binding residues were constructed in addition to a combination mutant of all four residues (Fig 3ii). Mutant proteins were expressed in *E*. *coli*, purified and structurally validated by circular dichroism and thermal shift analysis to ensure the mutations did not destabilise or influence protein structure (S2 Fig). Binding analysis indicated that each of the SElX single site-directed mutants of E154A, K156A, Q169A and the combination mutant SElX-EKQD-A exhibited almost complete loss of binding to both neutrophils and monocytes compared to the wild-type SElX protein, indicating that each of these 3 residues are required for SElX-mediated neutrophil and monocyte binding (Fig 3iii). The mutant SElX-D161A did not demonstrate a reduction in neutrophil or monocyte binding compared to wild-type SElX, indicating that this residue is not required for neutrophil or monocyte binding (Fig 3iii). Taken together these data demonstrate that the predicted sialic acid-binding motif of SElX is essential for binding to both human neutrophils and monocytes.

### SElX interacts with multiple neutrophil glycoprotein receptors

**Fig 4:**
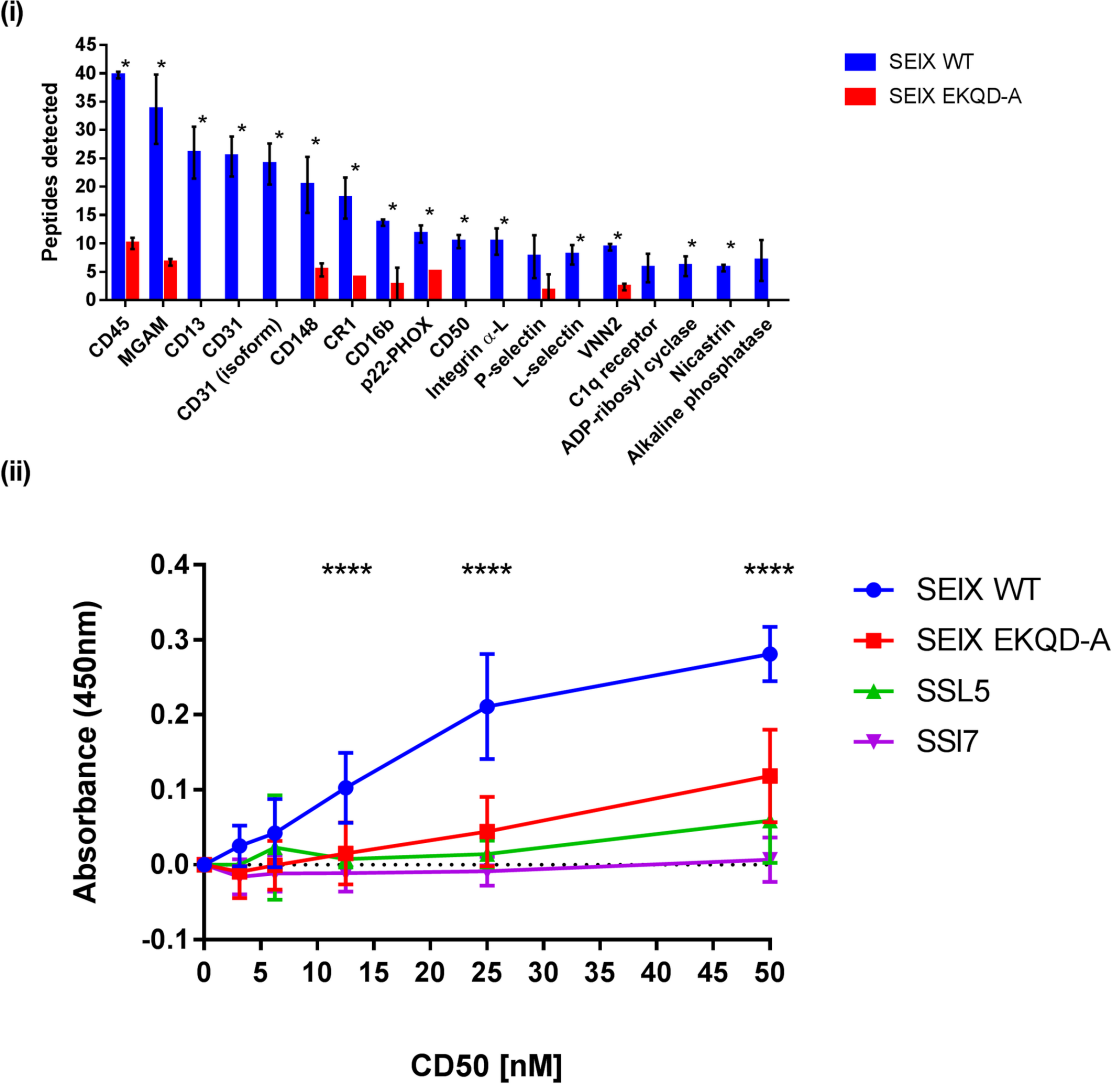
SElX binds to multiple neutrophil surface glycoproteins in a sialic acid-dependent manner. (i) Neutrophil lysates were analysed for peptide enrichment following incubation with wild-type SElX (WT) and SElX EKQD-A. Data shown indicates the mean number of peptides detected from each enriched protein (± SD from three donors). * denotes significance difference (p-value <0.05) in peptide enrichment between the wild-type and mutant protein as determined by multiple t-test comparisons (one unpaired, two tailed test per protein), without assuming similar standard deviation. (ii) ELISAs were performed comparing the interaction between staphylococcal proteins and recombinant CD50. Two-way ANOVA statistical analyses were performed. **** denotes a significant difference between SElX and SElX EKQD-A.

In order to identify the receptors for SElX-binding on the surface of neutrophils, affinity precipitation analysis of recombinant SElX and the neutrophil binding-deficient SElX-EKQD-A mutant was carried out with human neutrophil lysates, followed by quantitative mass spectrometry (MS) analysis. At least 12 proteins were enriched 5-fold or higher on SElX-coated Ni-NTA beads compared to Ni-NTA beads coated with SElX EKQD-A mutant protein suggesting that SElX binds to many neutrophil receptors in a sialic-acid dependent manner (S1 Table; Fig 4i). To test the hypothesis that individual neutrophil receptors could support binding of SElX we performed an ELISA-type assay with recombinant SElX and recombinant CD50 (ICAM-3), a protein that was enriched in the affinity precipitation analysis (Fig 4ii). The data demonstrated that SElX could bind directly to immobilized CD50 in a manner dependent on the sialic-acid binding motif (Fig 4ii).

All of the cell surface proteins identified to bind SElX are glycoproteins, consistent with the requirement for sialic-acid binding and represent an array of functional pathways in the neutrophil. Of note, the most enriched proteins are glycoproteins that display integrin or cell activation functions (CD45, CD13, CD31, CD50 and CD148) [24–27]. In addition, several cytosolic or granule-associate proteins were identified to bind to SElX, including p22-PHOX and the enzyme maltase-glucoamylase (MGAM) (found in the gelatinase and ficolin granules of neutrophils), both of which are involved in microbicidal functions of neutrophils [28, 29].

### SElX inhibits phagocytosis and killing by human neutrophils in a manner independent of its superantigenic function

**Fig 5:**
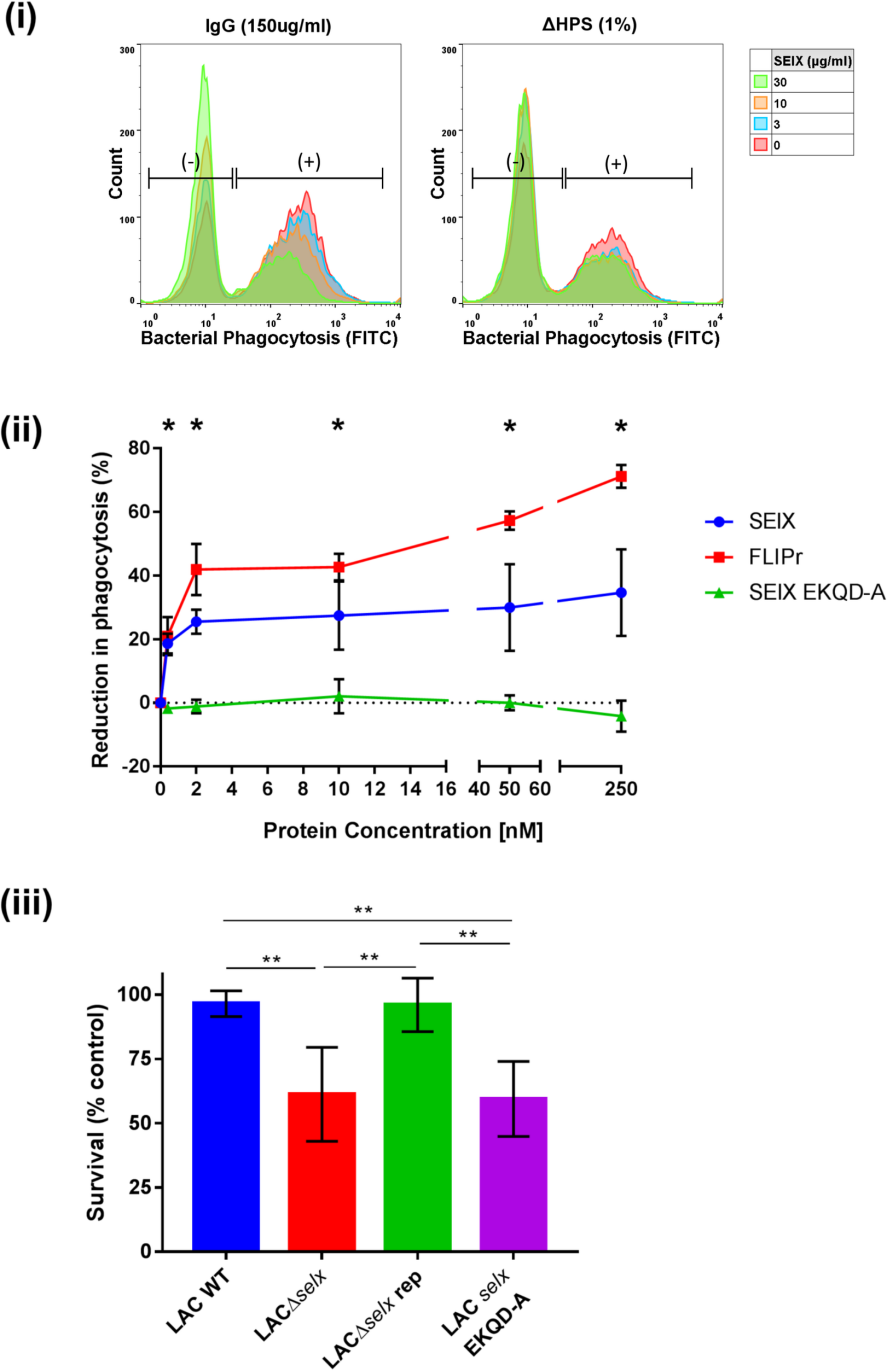
SElX inhibits IgG-mediated neutrophil phagocytosis and reduces killing by human neutrophils. (i) Phagocytosis of fluorescent-labelled *S*. *aureus* opsonized with 150 μg/ml of purified human IgG or 1% (v/v) complement-inactivated human pooled serum (ΔHPS), in the presence of SElX at various concentrations. Gates indicate the populations of neutrophils that have phagocytosed bacteria or not (+/-). (ii) Inhibition by recombinant SElX protein was compared to the IgG-mediated phagocytosis inhibitor FLIPr and the neutrophil-binding deficient mutant SElX EKQD-A. Phagocytosis was calculated as the percentage of cells with fluorescent bacteria and expressed relative to buffer-treated cells with 75 μg/ml human IgG. Results shown are the means of three different human donors (error bars SE of mean). Results between SElX wild-type and SElX EKQD-A protein were tested by two-way ANOVA and found to be significantly different, *indicates at which concentration a significant difference in phagocytosis was observed. (iii) *S*. *aureus* USA300 wild-type, *selx* deletion and site-directed mutants were incubated with isolated human neutrophils for 60 min. Following incubation neutrophils were lysed with Triton-X 100 and surviving bacteria plated and enumerated. Killing was calculated as the difference between the no neutrophil control and the surviving CFU. Data shown are the mean % surviving bacteria incubated with cells from 5 donors ± SD. CFU data were tested by students t-test (unpaired, two-tailed) with Welches correction, (* indicates a p value < 0.05, ** indicates a p value < 0.01).

To determine if SElX inhibits neutrophil-mediated phagocytosis of *S*. *aureus*, neutrophils were pre-incubated with SElX before the addition of opsonised bacteria. SElX reduced the ability of neutrophils to take up bacteria when heat-inactivated human pooled serum (ΔHPS) or IgG alone were applied as opsonins (Fig 5i). These data demonstrate that IgG-mediated phagocytosis is inhibited by SElX independently of the complement-mediated phagocytic pathway. SElX exhibits a highly potent activity at a concentration as low as 20 nM with a reduction in phagocytosis of up to 30%, compared to up to 70% for the Fcγ antagonist FLIPr (Fig 5ii). Of note, no reduction in phagocytosis was observed when the SElX EKQD-A mutant was pre-incubated with neutrophils (Fig 5ii), consistent with a requirement for sialic acid-binding dependent neutrophil interactions for phagocytosis inhibition.

To test the hypothesis that SElX enhances *S*. *aureus* survival in the presence of neutrophils, *S*. *aureus* strain USA300 LAC, a derivative *selx* deletion mutant, or a derivative that produces SElX-EKQD-A (Table 1, see S3 Fig for mutant validation) were each incubated with human neutrophils. The USA300 LAC parent strain was highly resistant to neutrophil killing consistent with previous reports [30, 31], but the *selx* deletion mutant and EKQD-A-expressing derivatives both exhibited increased susceptibility to killing (Fig 5iii). Importantly, re-introduction of *selx* to the LACΔ*selx* mutant restored killing resistance to wild-type levels (Fig 5iii). These data indicate that SElX enhances the capacity of *S*. *aureus* to survive neutrophil-mediated killing.

**Table 1:** Bacterial strains used in this study.

Strain	Description	Source/Reference
***S*. *aureus***		
USA300 LAC	Wild-type, Erythromycin (ERM) sensitive	[32]
USA300 LACΔ*selx*	Deletion of *selx*, ERM sensitive	[6]
USA300 LACΔ*selx* repaired (rep)	Reintroduction of *selx*, ERM sensitive	[6]
USA300 LAC *selx* EKQD-A	Re-introduced selx, with site-directed mutations of the sialic acid-binding motif, ERM sensitive	This Study
USA300 *spa*::Tn	Transposon insertion into *spa*	[33]
***E*. *coli***		
DH5α	Cloning strain	Invitrogen, Paisley, UK
BL21 DE3	Expression strain	Invitrogen, Paisley, UK
Strataclone SoloPack	*lacZΔM15* mutation, *endA*, *recA*-deficient	Agilent Technologies, UK

**Fig 6:**
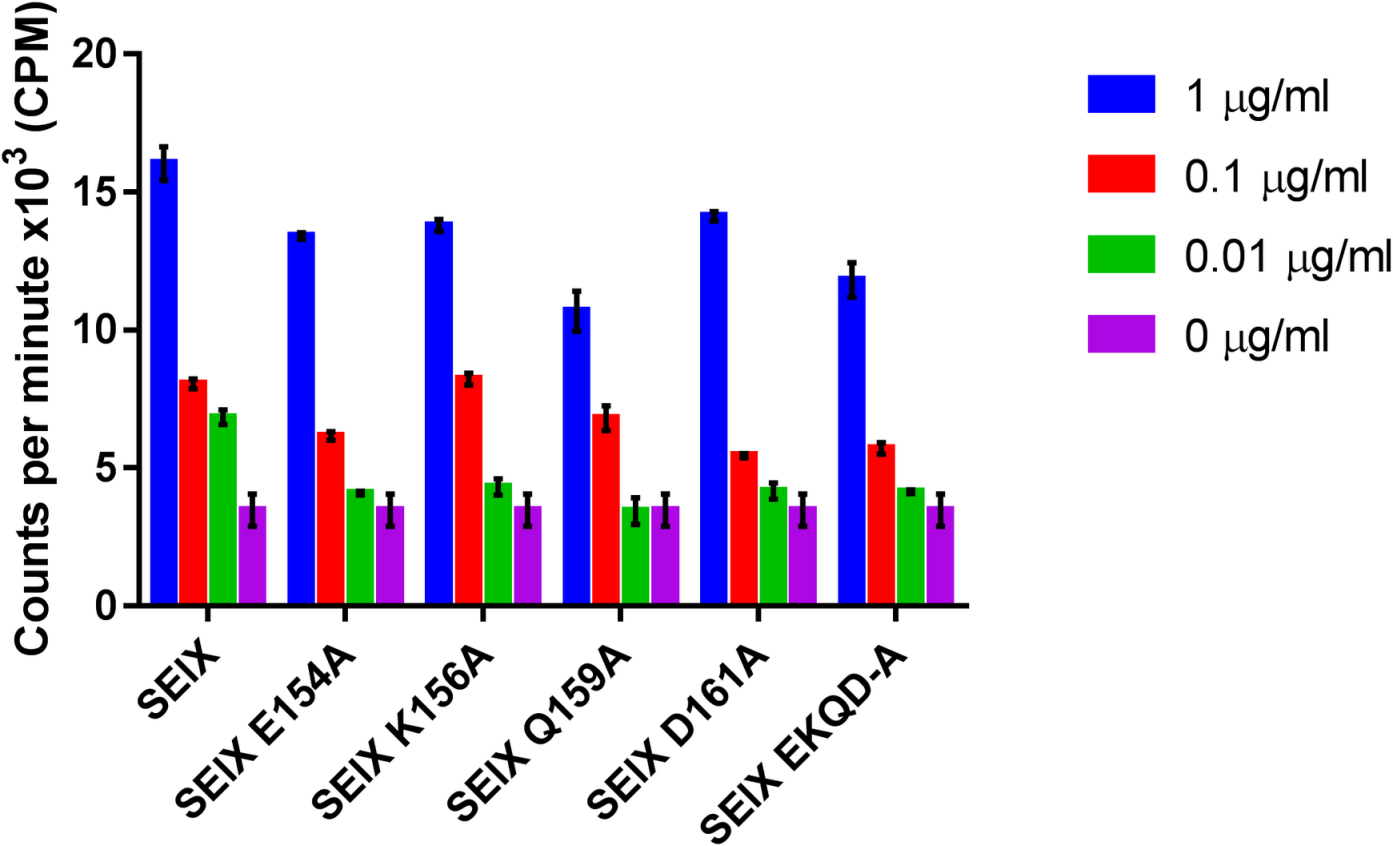
Neutrophil binding-deficient mutants of SElX retain mitogenic activity. Isolated human PBMC were stimulated with recombinant SElX and sialic acid-binding mutants. After 72 h incubation, proliferation was determined by analysing the incorporation of [^3^H] thymidine. Results shown are the means of triplicate measurement from 3 human donors ± standard deviation of the mean.

To determine if the superantigenic activity of SElX is distinct from its capacity to bind to neutrophils and inhibit phagocytosis, sialic acid-binding mutants of SElX were examined for their ability to induce T-cell proliferation by [^3^H] thymidine incorporation (Fig 6). All 5 mutants were capable of inducing T-cell proliferation at comparable levels inferring that the residues essential for neutrophil binding are not required for superantigenic activity. These data indicate that SElX exhibits two independent mechanisms of immune modulation affecting distinct cell types.

### SElX does not contribute to the severity of murine skin abscess infections

**Fig 7:**
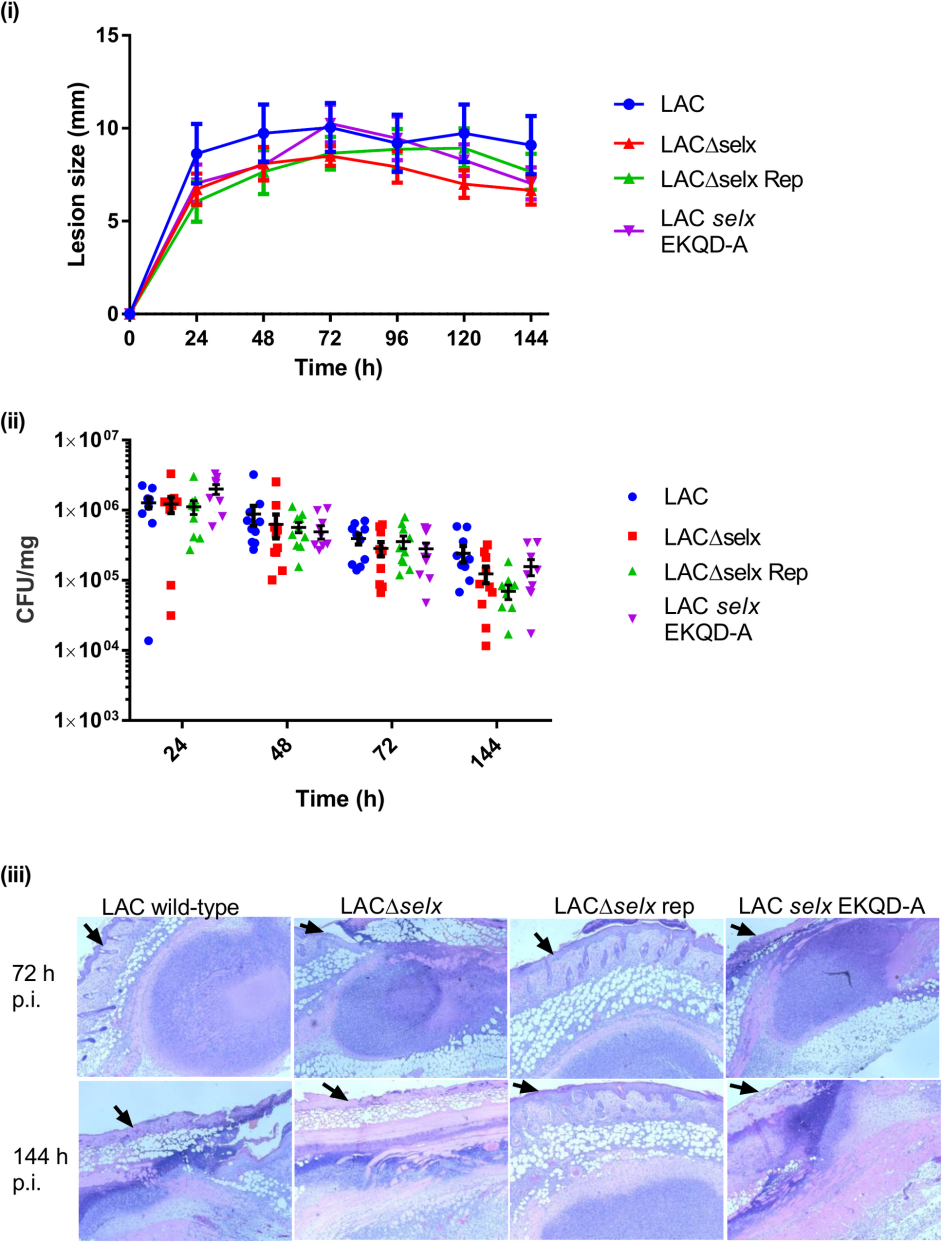
SElX does not contribute to *S. aureus* virulence in a mouse skin abscess infection model. (i) Lesion size of each mouse (n = 10) was measured every 24 h post inoculation (p.i.) for 6 d. Mean lesion size ± SEM is plotted for each of the 4 USA300 LAC mutant groups. (ii) Bacteria were recovered from excised skin lesions and enumerated by serial dilutions. CFU were normalised to the weight of tissue homogenised to give the bacterial load per mg of tissue. CFU per mg are displayed for each infected animal; the horizontal line indicates the mean CFU/mg of tissue and vertical bars show the SEM for each group (n = 10) (iii) Representative images from histological examinations of skin lesions 72 h and 144 h post inoculation. Mounted sections were stained with haematoxylin and eosin. Black arrows on each image indicate the surface of the epidermis.

To examine the role of SElX-neutrophil binding in the pathogenesis of skin infections, we carried out experimental murine skin abscess infections with USA300 LAC and derivative mutant strains (Table 1). Lesions developed within 24 h post injection and generally reached peak size within three days. No differences were seen in lesion size between the different mutant groups and the number of bacteria recovered at each time point declined over the course of the study up to 144 h (Fig 7). At each time point there were no differences seen in bacterial load between the different mutant and wild-type groups (Fig 7). For histopathological analysis, 6 mice were analysed from each experimental cohort and infected animals were sacrificed at 72 and 144 h post infection. Processed tissue slides were assessed for abscess morphology, severity of tissue damage and leukocyte infiltration. Diversity was observed in terms of both lesion morphology and severity although these features were not associated with a particular bacterial genotype (Fig 7). High levels of leukocyte infiltrations were observed in all slides but no differences detected among the different mutants groups in comparison to LAC wild-type infected tissue (Fig 7). Taken together, these data indicate that *selx* does not contribute to virulence in this murine skin abscess infection model.

### SElX-associated virulence in a rabbit model of necrotising pneumonia is dependent on the sialic-acid binding residues

**Fig 8:**
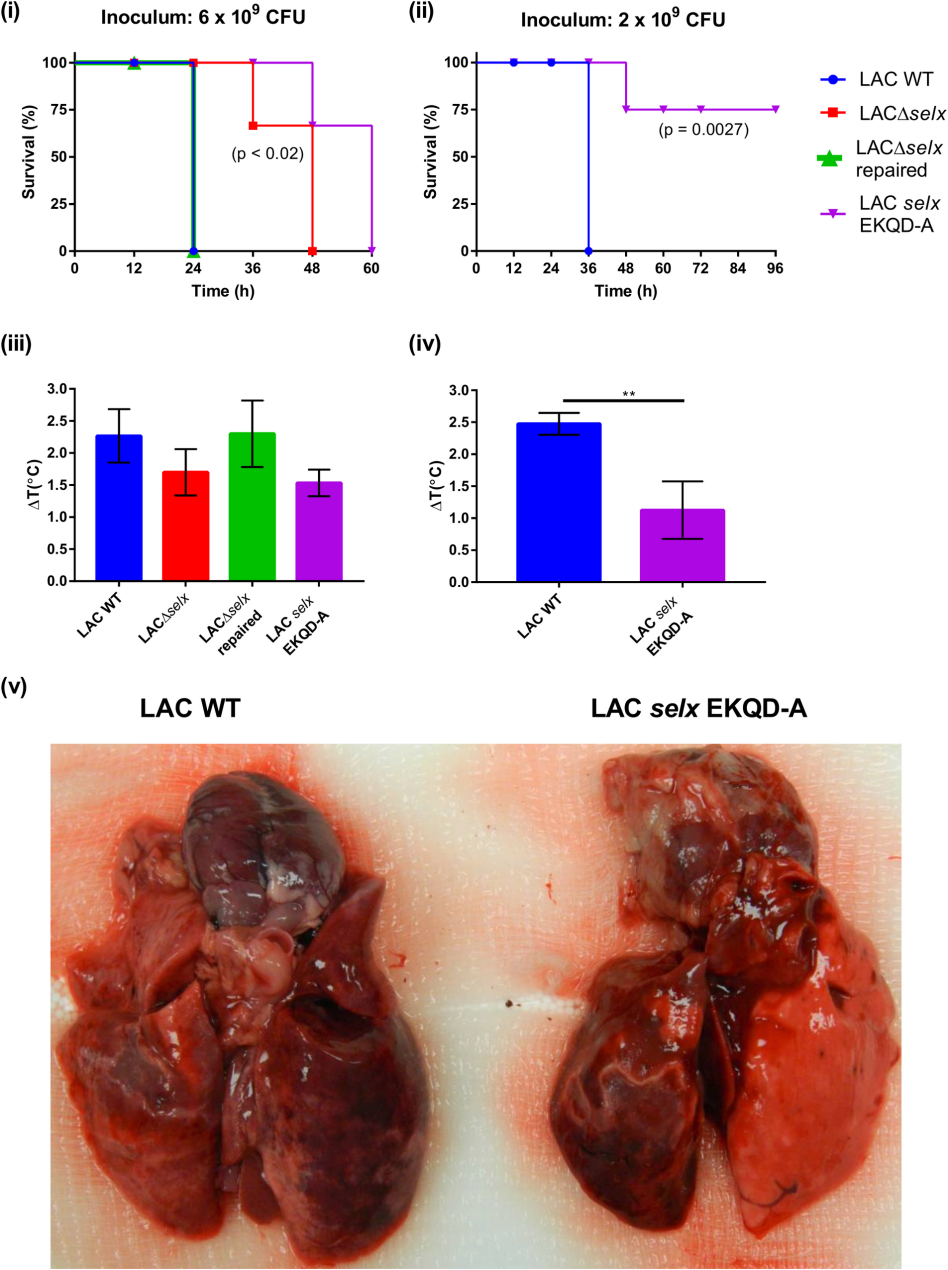
SElX contributes to lethality in a rabbit model of necrotising pneumonia by inhibiting neutrophil function. Kaplan-Meier curves of % survival of rabbits infected with wild-type *S*. *aureus* LAC, LACΔ*selx*, LACΔ*selx* rep and LAC *selx* EKQD-A at a dose of 6x10^9^ CFU (i) and *S*. *aureus* LAC and LAC *selx* EKQD-A at a dose of 2x10^9^ CFU (ii), p-values stated are the results of log-rank (Mantel-Cox) tests. (iii) Increase in rabbit core temperature (ΔT°C) 12 h after pulmonary infection with LAC, LACΔ*selx*, LACΔ*selx* rep and LAC *selx* EKQD-A at a dose of 6x10^9^ CFU (data plotted are the mean of three animals ± SD). (iv) Increase in rabbit core temperature (ΔT°C) 12 h after pulmonary infection with LAC and LAC *selx* EKQD-A at a dose of 2x10^9^ CFU (data plotted are the mean of four animals ± SD, ** indicates statistical significance by unpaired, two-tailed students t-test p<0.003) (v) Gross pathology of lungs from rabbits infected with *S*. *aureus* strain LAC and LAC *selx* EKQD-A showing representative examples of haemorrhagic lesions.

Previously, SElX has been demonstrated to contribute to lethality in a rabbit model of necrotising pneumonia [6]. Here, the experiment was repeated including the *selx*-EKQD-A-expressing USA300 LAC to examine the relative contribution of superantigenicity and neutrophil-binding functions of SElX to the outcome of infection. At an inoculum of 6 x 10^9^ CFU, rabbits infected with LACΔ*selx* or LAC *selx* EKQD-A had longer survival times (Fig 8i) and had less elevated body temperatures than animals infected with wild-type LAC or repaired strain (LACΔ*selx* rep) (Fig 8iii). Similarly, at a lower inoculum of 2 x 10^9^ CFU animals challenged with the LAC *selx* EKQD-A mutant strain had increased survival (p = 0.0027; Fig 8ii) and less elevated body temperature compared to animals infected with the wild-type (p = 0.0014; Fig 7iv). On gross examination, lungs from rabbits infected with wild-type LAC were dark red to purple indicating severe haemorrhage (Fig 8v left). In contrast, lungs from rabbits infected with the LAC *selx* EKQD-A mutant strain demonstrated signs of haemorrhage in one lobe but tissue was relatively healthy in the second lobe (Fig 8v right). Taken together, these data indicate that residues essential for sialic acid-binding of SElX are required for the pathology caused by SElX in a rabbit model of necrotising pneumonia. As a role for SElX in pathogenesis in a murine skin abscess model was not observed in the current study, these data highlight important host- or tissue-specific differences relevant to the choice of infection model employed for investigating *S*. *aureus* pathogenesis.

## Discussion

As previously reported in Wilson et al (2011), SElX shares sequence homology with SAgs and SSls, suggesting that SElX may have properties of both families of proteins. In this study we have demonstrated that SElX exhibits characteristics of both groups and can therefore be described as a functional hybrid of the SAgs and SSls. SElX is a bi-functional protein that binds to neutrophils and inhibits IgG-mediated phagocytosis via a mechanism that is distinct from its superantigenic activity. This is the first time that bi-functionality has been observed in superantigens independent of the emetic activity of staphylococcal enterotoxins. The common multifunctionality of *S*. *aureus* virulence factors is an emerging theme in *S*. *aureus* pathogenesis research. For example, SSl5 binds to neutrophils via PSGL-1 and also inhibits the function of metalloprotease 9 resulting in the inhibition of cell activation by chemokines, rolling and migration of neutrophils to the site of infection, and reduction in the formation of thrombi [34–38]. In addition, Staphylococcal protein A (SpA) subverts opsonisation by binding the Fc region of IgG molecules and can also act as a SAg for B-lymphocytes leading to disruption of the humoral immune response [39]. Cytolytic toxins from *S*. *aureus* (including Hla, HlgACB, PVL and LukAB) in addition to their cytolytic activity on haematopoietic cells have been shown at sub-lytic concentrations to activate the intracellular NOD-like Receptor protein 3 (NLRP3) inflammasome in neutrophils, monocytes and macrophages which lead to pro-inflammatory cytokine release [21, 40–44]. Of note, we have identified a protein that employs distinct mechanisms to target two distinct immune cell types linked to innate and acquired immune responses, respectively.

The SaeRS two-component system controls an array of *S*. *aureus* virulence factors such as CHIPS, SCIN, the SSls, and SAgs including TSST-1, which are involved in immune evasion in response to host stimuli [45–47]. Transcriptomic analysis of a USA300 deletion mutant of SaeRS, performed by Nygaard et al, resulted in almost a five-fold decrease in the transcription levels of *selx* (SAUSA300_0370) [47]. These data suggest that *selx* is regulated by SaeRS in co-ordination with an array of other immune modulators. Considering its dual modes of function, SElX is likely produced by *S*. *aureus* early in infection in response to neutrophil signals but may persist at the site of infection as the adaptive immune response is recruited, leading to induction of T-cell proliferation and further immune dysregulation. Alternatively, SElX activation of lymphocytes resulting in the release of cytokines and chemokines (such as interferon-γ and interleukin 6) may stimulate the recruitment of neutrophils, as reported previously for SEA [48]. As neutrophils are recruited to form an abscess, SElX may enhance bacterial survival by inhibiting phagocytosis and stimulating misdirecting cytokines from lymphocytes which could also inhibit neutrophil function. We observed that SElX exhibited low levels of binding to human lymphocytes and this was limited to CD4^+^ and CD8^+^ T-cells with no binding to B-lymphocytes (S1 Fig). This is a characteristic of the SSl-proteins that bind to human leukocytes and may reflect the low activity of glycosyl-transferases in peripheral lymphocytes prior to activation [49]. In lymphocytes, glycosyl-transferases are activated by the presence of IL-2, an interleukin induced during SAg-mediated activation of the lymphocyte [49]. It is possible that SElX induces glycosylation on the surface of lymphocytes through SAg-mediated activation and then acts as a glycoprotein antagonist modulating lymphocyte function. However, further experiments would be required to investigate this hypothesis.

SElX demonstrates the ability to interact with neutrophils from multiple mammalian species including humans, rabbits, mice, and cattle. Dairy cows and farmed rabbits represent important veterinary hosts of *S*. *aureus* [50] and the ability of SElX to bind to neutrophils from these species, in addition to humans, suggests a broad role for SElX in pathogenesis of *S*. *aureus* mammalian hosts (Fig 1). Previous studies of *S*. *aureus* virulence have demonstrated that rabbits may represent more appropriate infection models than mice for the analysis of some virulence factors such as Panton-Valentine leucocidin (PVL) [51]. Although we identified that SElX bound strongly to murine neutrophils, we did not detect any SElX-dependent effect on virulence in a murine skin abscess model (Fig 7). This may reflect low levels of SElX expression during murine skin infection or alternatively it may be due to the previously observed differential glycan decoration on murine cell receptors compared to humans [52–55], which could impact on the capacity of SElX to bind to specific target receptors with downstream pathogenic consequences.

Our data suggest that the effect of SElX on neutrophils is independent of cytolysis and apoptosis (S4 Fig) and that it can mediate an inhibition of IgG-mediated phagocytosis, consistent with its capacity to bind to the Fcγ receptor CD16b. However, the observed promiscuous binding of SElX to an array of glycosylated neutrophil proteins suggests that SElX may disrupt other neutrophil functions. For example, another SElX binding partner ICAM-3 (CD50) is an important signalling molecule associated with the lymphocyte function-associated antigen 1 (LFA-1) which was also identified as a ligand for SElX (α-L integrin) [56, 57]. In addition, several proteins linked to the phagosome including MGAM (a glucamase present in neutrophil granules) [29] and p22-PHOX (a key component of the cytochrome B complex) [28] were able to bind to recombinant SElX in a sialic acid dependent manner (Fig 4), suggesting that SElX may interfere with neutrophilic enzymes involved in phagosomal killing.

The multiple functions exhibited by many *S*. *aureus* virulence factors contribute to the remarkable apparent redundancy of function promoting the evasion of the immune response. This is an important consideration for the development of vaccines and anti-virulence therapy as therapeutic or preventive measures targeting a small number of virulence factors may prove ineffective if similar functions can be mediated by alternative proteins. Therefore, developing an understanding of the full functional repertoire of *S*. *aureus* virulence factors will inform the rational design of targeted therapies that by-pass the intrinsic redundancy in *S*. *aureus* pathogenesis.

In conclusion, we have demonstrated that SElX is a bi-functional SAg with distinct modulatory effects on critical functions of the innate and acquired immune response. Given the large family of SAgs identified to date, our findings imply that further investigations into the alternative functions of SAgs are warranted.

## Material and methods

### Ethics statement

Human venous blood was taken from healthy donors in accordance with a human subject protocol approved by the national research ethics service (NRES) committee London City and East under the research ethics committee reference 13/LO/1537. Volunteers were recruited by a passive advertising campaign within the Roslin Institute (University of Edinburgh) and following an outline of the risks, written informed consent was given by each volunteer before each sample was taken. De-identified human blood packs from consenting-healthy individuals were also purchased from the New York Blood Center. The New York City Blood Center obtained written informed consent from all participants involved in the study and risks were outlined to each donor prior to sampling. The use of the de-identified samples was reviewed and approved by the New York University School of Medicine University Committee on Activities Involving Human Subjects.

All experiments using animals were handled in strict accordance with good animal practice as defined by the relevant national and local animal welfare bodies. Specifically, experimentation using rabbits were performed under a University of Iowa approved Institutional Animal Care and Use Committee (IACUC) protocol (4071100). Animals were maintained in accordance with the Animal Welfare Act (1966) (administered by the US Department of Agriculture) and the U.S. Department for Health and Human Services (DHHS) ‘‘Guide for the Care and Use of Laboratory Animals”. University of Iowa is accredited by the Association for Assessment and Accreditation of Laboratory Animal Care International (AALAC). For intra-pulmonary administration of bacterial strains, animals were anesthetized with use of ketamine (10 mg/kg) combined with xylazine (10 mg/kg). In agreement with the University of Iowa IACUC, animals that failed to exhibit escape behaviour and at the same time could not right themselves were prematurely euthanized according to predetermined experimental endpoints. Animals were euthanized with intravenous injection of 2 ml of Euthasol, whether prematurely or at the end of experimentation. Euthanized animals were then subjected to bilateral thoracotomy to ensure euthanasia. All animals received pain-relieving medication (buprenorphine; 0.05 mg/kg intramuscular twice daily) for the duration of experimentation.

All murine experiments were carried out under the authority of a UK Home Office Project License (PPL 70/08663) within the terms and conditions of the regulations of the UK Home Office Animals (Scientific Procedures) Act 1986 and the code of practice for the housing and care of animals bred, supplied, or used for scientific purposes. The study protocol was reviewed and approved by the Roslin Institute Small Animal Review Group (University of Edinburgh) which includes the Named Veterinary Surgeon (NVS), Named Animal Care and Welfare Officers (NACWOs) and a statistician services prior to each experiment. Animals were monitored twice daily to ensure that no animal exceeded predetermined severity agreed euthanasia criteria according to the study protocol. Animals were euthanized, whether prematurely or at the end of experimentation by asphyxiation using carbon dioxide. Euthanized animals were then subjected to cervical dislocation to ensure euthanasia.

Chicken immunisation was provided by the Scottish national blood transfusion service (Pentland Science Park, Midlothian, UK) using unembryonated hen’s eggs. Procedures performed were carried out under the authority of a UK Home Office Project License (PPL 60/4165) within the terms and conditions of the regulations of the UK Home Office Animals (Scientific Procedures) Act 1986 and the code of practice for the housing and care of animals bred, supplied, or used for scientific purposes.

### Bacterial strains and culture conditions

*S*. *aureus* strains were cultured in tryptone soya broth (TSB) or brain heat infusion broth (BHI) (Oxoid, UK) with shaking at 200rpm, or on tryptone soya agar (TSA) (Oxiod, UK) at 37°C for 16 h unless otherwise stated. *E*. *coli* strains were cultured in Luria broth (LB) (Melford laboratories, UK) with shaking at 200rpm, or on LB-agar (Melford laboratories, UK) at 37°C for 16 h unless otherwise stated. Media was supplemented, where appropriate, with antibiotics. Strains were stored in the appropriate liquid culture media containing 40% (v/v) glycerol (Sigma-Aldrich, UK) in cryovials (Nunc, Thermo Scientific, UK) at -80°C.

### Leukocyte isolation

50 ml of venous blood was drawn from healthy human volunteers and mixed with 6 ml of acid-citrate-dextran (ACD) (25 g D-glucose (Sigma-Aldrich, UK) and 20.5 g trisodium citrate (Sigma-Aldrich, UK) added to 1 L of ddH_2_O). Human neutrophils and peripheral blood mononuclear cells (PBMC) were isolated as outlined previously [58] and re-suspended in RPMI1640 (Gibco, UK) containing 0.05% human serum albumin (HSA) (Sigma Aldrich, UK) for further analysis.

Murine bone marrow derived neutrophils were isolated from femurs of wild-type mice from BALB/C backgrounds, as described previously [59]. Neutrophil cell identity was confirmed using Lys6C and Lys6G staining by incubating the isolated cells with 1:500 dilutions of Rat anti-Lys6C PerCP-Cy5.5 (AL-21; BD Bioscience, Oxford, UK) and Rat anti-Lys6G PE-Cy7 (1A8; BD Bioscience, Oxford, UK) for 30 min on ice and then analysed by flow cytometry (FACSCalibur; Becton Dickinson, Franklin Lanes, NJ). Expression of neutrophil cell surface markers was confirmed by a high fluorescent signal for Lys6G and Lys6C.

Bovine neutrophils were isolated from Holstein-Friesian cattle aged 18 to 36 m via jugular vein puncture (6 ml 10 x PBS/EDTA (100 mM KH_2_PO_4_, 9% (w/v) NaCl, and 2 mg/l EDTA in sterile H_2_O adjusted to pH 7.4) anticoagulant used for 50 ml of blood). Neutrophils were isolated from bovine blood using a previously described protocol [60].

Rabbit neutrophils were isolated from peripheral rabbit blood (using ACD as an anticoagulant) purchased from the National Transfusion Centre, Moredun Institute, Midlothian, UK. Leukocytes were isolated using an equal volume of red blood cell (RBC) lysis buffer (10 x buffer contained; 155 mM NH_4_Cl, 10 mM KHCO_3_ and 100 μM EDTA in ddH_2_O). RBCs were lysed in 1x lysis buffer for 10 min at 37°C and the remaining cells were pelleted at 400 x g for 10 min and washed with HBSS (Mg^2+^ and Ca^2+^ free) (Gibco) and this was repeated until the pellet was clear of RBCs. All cells were counted with a TC20 automated cell counter (Biorad, Hemel Hempstead, UK) and re-suspended to a concentration of 1 x 10^7^ cells/ml in the desired assay media/buffer.

### Western blot analysis

Liquid cultures of *S*. *aureus* strains were grown in BHI broth to stationary growth phase (OD_600_ = 4.0–7.0). Cultures supernantants were concentrated using Amicon Ultra-15 Centrifugal Filter units (10000 MWCO) (Millipore, Watford, UK). Concentrated secreted proteins were separated by sodium dodecyl sulfate polyacrylamide gel electrophoresis (SDS-PAGE, 12% acrylamide gel) and transferred to nitrocellulose membranes using the Trans-Blot Turbo Blotting system (Bio-Rad, Hemel Hempstead, UK), according to the manufacturer’s specifications. The membrane was incubated in 1 x PBS (pH 7.3) containing 8% (w/v) powdered milk (Sigma-Aldrich, UK), at 4°C overnight. The membrane was incubated with primary antibody (anti-SElX chicken IgY), at a dilution of 4μg/ml for 2 h. Anti-SElX IgY was obtained from immunised unembryonated hen’s eggs using the Eggspress IgY purification kit (Gallus Immunotech, NC, USA) according to the manufacturer’s instructions. Following primary antibody incubation the membrane was washed 3 times with washing buffer, 1 x PBS (pH 7.3) containing 1% (w/v) powdered milk and 0.05% (v/v) Tween 20 (Sigma-Aldrich, UK). The membrane was incubated with a horse radish peroxidase-conjugated (HRP) secondary antibody (goat anti-chicken-IgY IgG (Source Bioscience, Nottingham, UK)), at 0.5 μg/ml in washing buffer, for 1 h, followed by a further 3 washes and then incubated with ECL reagent for 5 min. The blot was then exposed on Hybond ECL film (Amersham, Systems, Buckinghamshire, UK) for 20 s and developed using an X-ray developer (SRX-101A, Konic Minolta, Japan).

### Recombinant protein expression

*E*. *coli* expression strain BL21 DE3 containing plasmid constructs (pET15b::*selx2*, pET15b::*ssl7* [6], and pRSETB::*ssl5* [12]) for protein expression were cultured in LB containing selective antibiotics and induced in mid-exponential phase of growth (OD_600_ = 0.6), with 1 mM isopropyl b-D-1-thiogalactopyranoside (IPTG) (Formedium Ltd., Norfolk, UK) for 4 h at 37°C. Cells were recovered by centrifugation at 4000 x g, disrupted using a One-Shot cell disruptor (Constant Systems, Northants, UK), and His-tagged recombinant proteins were purified by affinity chromatography on a His-Trap FF crude nickel affinity column (GE healthcare, Buckinghamshire, UK) using an AKTA fast protein liquid chromatography (FPLC) OPC900 P920 system (GE Healthcare, Buckinghamshire, UK). SSl5 was purified using the buffer system outlined previously [9]. SSl7 purification was performed using a buffer containing 50 mM NaH_2_PO_4_, 300 mM NaCl and 10 mM imidazole (pH 8.0). For washing, imidazole concentration was increased to 20mM and a concentration of 250 mM imidazole was used for elution. SElX was purified under denaturing conditions in a lysis buffer of 100 mM NaH_2_PO_4_, 10 mM Tris•Cl and 8 M urea (pH 8), the protein was washed with the same buffer at pH 6.3 then eluted at a pH of 4.5. To further purify SElX, ion exchange chromatography was used by diluting the affinity chromatography elution (1/10) in 50 mM HEPES (pH 7.5) and binding it to a Hitrap SP FF column. Protein was eluted over a NaCl gradient. Purified proteins were dialysed in 1 x PBS (pH 7.3) using Spectra/Por Float-A-Lyzer tubing with an 8000 to 10000 Da molecular weight cut off (MWCO) (Spectrum Laboratories, CA, USA). Proteins were quantified using a Nano-Drop ND1000 spectrophotomer (Thermo scientific, USA) set on the A280 program. After the protein solution spectra were obtained, the concentration of the protein was calculated at an absorbance of 280 nm and the extinction coefficient calculated from the protein sequence as described previously [61].

### Flow cytometry binding analysis

For binding of recombinant proteins to leukocytes, neutrophils (5 × 10^6^ cells/ml) and PBMCs (5 × 10^6^ cells/ml) were incubated with increasing concentrations of 6 x HIS-tagged recombinant proteins in RPMI 1640 (Gibco, UK) and 0.05% HSA (human serum albumin (Sigma-Aldrich, UK), RPMI-HSA) for 30 min on ice. For some leukocyte binding experiments the cells were also co-incubated with cell surface subset–specific antibodies including anti-CD4 (Leu-3a), -CD8 (Leu-2a), and, -CD19 (Leu-12) (PE labelled) (BD bioscience, Oxford, UK). Cells were washed with RPMI-HSA and pelleted at 400 x g for 10 min at 4°C. Binding of the proteins was detected with a FITC-labelled monoclonal mouse anti-HIS tag monoclonal antibody at a 1.25 μg/ml final concentration (AD1.1.10; LS Bioscience, WA, USA), antibodies were incubated with cells for 30 min on ice, washed with RPMI-HSA and pelleted at 400 x g for 10 min at 4°C. Following washing, cells were re-suspended in 200 μl of RPMI-HSA and leukocyte populations were identified based on forward and sideway scatter on a BD FACSCaliburflow cytometer (Becton Dickinson, Franklin Lanes, NJ), and fluorescence was measured. To analyse leukocyte apoptosis, cells were washed one additional time following the binding experiment and re-suspended in assay media containing the nuclear dye DRAQ 5 (Biostatus, UK) (diluted 1/250) prior to flow cytometry analysis. For neuraminidase experiments, isolated leukocytes (2 x 10^6^ cells/ml) were pre-treated with 0.2 U/ml neuraminidase (from *Vibrio cholera*; Sigma-Aldrich, UK) at 37°C for 45 min at pH 6.0, prior to washing and subsequent incubation with proteins.

### Site directed mutagenesis

Site-directed mutagenesis was performed to exchange amino acids in the sequence of SElX with alanine by introducing mutations into the pET15b::*selx2* construct using PCR with oligonucleotide primers listed in Table 2. The reactions were performed using the PfuUltra II Fusion HS DNA polymerase (Agilent Technologies, UK). Primers were used at a final concentration of 250 nM along with 2 mM dNTPs (Promega, Hampshire, UK). PCR cycle conditions were as follows; 1 cycle at 95°C for 2 min, 30 cycles of 95°C for 20 s, 50°C for 20 s and 72°C for 90 s, followed by a final extension of 3 min at 72°C. Following the PCR reaction, *Dpn*I endonuclease (NEB, Herts, UK) was added to a final concentration of 0.8 U/μl, the reaction was then incubated for 1 h at 37°C followed by an enzyme deactivation step of 10 min at 65°C. Following digestion, 1 μl of the amplified vector product was transformed into *E*. *coli* Solopack cells from the Strataclone blunt cloning kit (Agilent technologies, UK) following the manufacturer’s instructions. Transformation plates were incubated overnight at 37°C and screened for transformed colonies, which were randomly selected for Sanger sequencing (Edinburgh Genomics, University of Edinburgh). When the mutations were confirmed the plasmid constructs were transformed into BL21 DE3 *E*. *coli* cells for protein expression and purification. Following the purification of the mutated proteins, analysis was performed to show the proteins had folded correctly and not become unstable. Far UV circular dichroism (CD) spectra of samples were acquired on a Jasco J-710 spectrometer (Japan Spectroscopic Co. Ltd, Japan). Spectra of the proteins, at a concentration of 0.4 mg/ml to 1 mg/ml in 0.1 x PBS (pH 7.3), was recorded between 190 nm to 250 nm using a cuvette with a path length of 0.05 mm. Thermal shift analysis was performed using SYPRO Orange dye (Life technologies, UK) and conducted in a Lightcycler 480 (Roche, West Sussex, UK) using the melt curve function from 25°C to 95°C. Solutions of 5 μM recombinant staphylococcal protein were mixed with SYPRO Orange dye diluted 1/125 of the stock dye. The plate was heated from 25°C to 95°C in temperature increments of 0.1°C/sec. The fluorescence intensity was measured with excitation/emission at 465/510 nm.

**Table 2:** List of primers used in this study.

Primer Name^a^^,^^b^	Sequence (5’-3’)	Nucleotide Mutation^c^	Amino Acid Exchange
Cloning Primers			
pMAD MCS F	GCAACGCGGGCATCCCGATG		
pMAD MCS R	CCCAATATAATCATTTATCAACTCTTTTACACTTAAATTTCC		
T7(pET MCS) F	TAATACGACTCACTATAGGG		
T7(pET MCS) R	GCTAGTTATTGCTCAGCGG		
*selx seq* F	AGGTATCATCTATGGGGGAACA		
*selx seq* R	ATGATGGTGCTAATCATAACAAAGA		
*selx out* F	ATGTGGCTAATTTTGTTCGAGTCG		
*selx* out R	CGTCATGCGTTACTTTCGTTCG		
Site-directed Mutagenesis			
E153 F	GTCATAAATACAAAAGATGGTGGTAAATATACATTAGCTTCGCATAAAGAGCTACAAAAAGATAGGG	499 GAG>GCT	E153A
E153 R	CCCTATCTTTTTGTAGCTCTTTATGCGAAGCTAATGTATATTTACCACCATCTTTTGTATTTATGAC		
K156 F	GATGGTGGTAAATATACATTAGAGTCGCATGCAGAGCTACAAAAAGATAGGGAAAAT	508 AAA>GCA	K156A
K156 R	ATTTTCCCTATCTTTTTGTAGCTCTGCATGCGACTCTAATGTATATTTACCACCATC		
Q159 F	GAGTCGCATAAAGAGCTAGCAAAAGATAGGGAAAATGTAAAAA	517 CAA>GCA	Q159A
Q159 R	TTTTTACATTTTCCCTATCTTTTGCTAGCTCTTTATGCGACTC		
D161 F	GAGTCGCATAAAGAGCTACAAAAAGCAAGGGAAAATGTAAAAATT	524 GAT>GCA	D161A
D161 R	AATTTTTACATTTTCCCTTGCTTTTTGTAGCTCTTTATGCGACTC		
EKQD F	GGTGGTAAATATACATTAGCTTCGCATGCAGAGCTAGCAAAAGCAAGGGAAAAT	499 GAG>GCT 508 AAA>GCA 517 CAA>GCA 524 GAT>GCA	E153A K156A Q159A D161A
EKQD R	ATTTTCCCTTGCTTTTGCTAGCTCTGCATGCGAAGCTAATGTATATTTACCACC		

^a^ F and R refer to forward and reverse primers respectively

^b^ Multiple cloning site (MCS)

^c^ Positions relative to *selx2* allele (NCBI gene accession: SAUSA300_RS01970)

To prepare an *S*. *aureus* construct containing the site-directed mutations in *selx* the same PCR protocol was applied to pMAD::*selx* rep [6] as described previously for pET15b::*selx2*. Following confirmation of the mutations by sequencing, pMAD::*selx* EKQD-A was transformed into *S*. *aureus* RN4220, then transduced into LACΔ*selx* using phage 80α [62]. To aid transduction and allelic replacement, all USA300 mutants were made erythromycin sensitive by serial passage on TSA. Following transduction, allelic replacement was performed as described previously [6].

### Genomic and phenotypic validation of mutants

Phenotypic analysis and whole genome sequencing was performed on USA300 LAC constructs (Table 1) to confirm no spurious mutations had occurred (S3 Fig). 250bp Paired End Illumina HiSeq 2500 reads were mapped to the USA300 FPR3757 (NC_007793) reference genome using various tools implemented in the Nesoni pipeline (https://github.com/Victorian-Bioinformatics-Consortium/nesoni). Genome sequence data has been deposited at the European Nucleotide Archive under study number PRJEB20077 with the accession numbers ERS1625171, ERS1625172, ERS1625173 and ERS1625174.

Growth curve analysis was performed on a Floustar Omega microplate reader (BMG Labtech, Germany). Overnight cultures of bacteria were diluted 1/100 in either TSB or BHI and growth was recorded over 18 h. SDS-PAGE analysis was performed on concentrated supernatants and cell wall extracts (CWA). To extract CWA proteins, pelleted cells were washed with 1 ml PBS (Oxoid, Cambridge, UK), re-suspended in 1 ml lysis buffer (50 mM TrisHCl, 20 mM MgCl2, 30% Raffinose (Fluka, UK), adjusted to pH 7.5) containing 200 μg/ml Lysostaphin (AMBI products LLC, NY, USA) and protease inhibitors (Roche, Switzerland) and incubated at 37°C for 20 min. Samples were centrifuged at 6000 x g for 20 min and CWA proteins were recovered from the supernatant fraction.

### *S*. *aureus* neutrophil killing assays

*S*. *aureus* were cultured to an OD_600_ of 4.0 in BHI broth, washed by diluting in PBS, followed by centrifugation at 4000 x g for 10 min. The cells were then diluted to 1x10^4^ CFU/ml in RPMI containing 10% (v/v) complement-inactivated serum (inactivated by incubation at 56°C for 30 min). Isolated human neutrophils were infected at a ratio of 1 bacterium to 1000 neutrophils, and incubated for 60 min at 37°C with vigorous shaking. 250 μl of the neutrophil bacterial suspension was diluted into 750 μl of PBS containing 0.05% (v/v) Triton-X 100 and incubated for 5 min at room temperature to lyse the neutrophils. A control reaction was also prepared; no neutrophils were added to the RPMI with heat inactivated serum and treated in the same way as the test samples. Viable bacteria in each reaction mixture were enumerated by serial dilution and plating on to TSA, followed by overnight incubation at 37°C. Surviving bacteria were counted and compared to the no neutrophil control to determine bacterial survival.

### Phagocytosis assay

Phagocytosis efficacy was measured using FITC-labelled *S*. *aureus* USA300 *spa*::Tn (Table 1), bacteria were labelled as outlined previously [63]. To determine the level of neutrophil phagocytosis, FITC-labelled *S*. *aureus* were mixed with complement-inactivated human serum or purified IgG for 15 min at 37°C in 2 ml 96-well v-bottomed plates (Corning, USA) to facilitate opsonisation. Subsequently, isolated human neutrophils, with or without recombinant staphylococcal protein (pre-incubated for 30 min at room temperature), were added at a 10:1 (bacterium/cell) ratio and incubated for 15 min at 37°C with shaking at 750 rpm. The reaction was stopped with 1% (v/v) paraformaldehyde (Fisher Scientific, UK), and cell-associated fluorescent bacteria were analysed by flow cytometry. Phagocytosis was defined as the percentage of cells with a positive fluorescent signal. Reduction in phagocytosis was calculated by normalising the percentage from test samples to that of uninhibited cells for each opsonin.

### Cytotoxicity determined by lactate dehydrogenase (LDH) release

Protein mediated cytotoxicity was determined on human neutrophils, this was analysed with 1x10^5^ cells/ml suspended in RPMI 1640 (Gibco, USA) supplemented with 10% heat-inactivated fetal bovine serum (Gemini Bio Products, CA, USA) at a concentration of 1x10^5^ cells/ml. Recombinant *S*. *aureus* proteins were added and incubated for 1 h at 37°C with 5% CO_2_. Following protein treatment, cells were pelleted by centrifugation at 450 x g at 4°C for 5 min. Lactate dehydrogenase (LDH) release was assayed as a measure of neutrophil viability using the CytoTox-ONE homogeneous membrane integrity assay (Promega, WI, USA) according to the manufacturer’s specifications. Fluorescence was measured using a PerkinElmer Envision 2103 multilabel reader (excitation, 555 nm; emission, 590 nm) (PerkinElmer, MA, USA), and data were normalized to 100% lysis as determined by the addition of 0.2% Trition X (v/v) to the neutrophils.

### Affinity precipitation experiments

Detergent-solubilized proteins from primary human neutrophils were incubated with 6 x HIS-tagged SElX and interacting proteins were purified using Ni-NTA resin (Qiagen, Manchester, UK). Proteins eluted from Ni-NTA resin were separated by SDS-PAGE (4% to 15% gradient gel) and stained with SYPRO-Ruby (Life Technologies, UK). Gels were imaged and the lanes were excised for trypsin digestion, protein extraction and liquid chromatography-tandem mass spectrometry (LC-MS/MS) analysis as described previously [64].

### Enzyme-linked immunosorbent assay (ELISA) analysis

96-well ELISA plates (Maxisorb; Nunc, Thermo, UK) were coated with 10 μg/ml recombinant SElX, SElX EKQD-A, SSL5 or SSl7 in carbonate/bicarbonate buffer (50 mM Na_2_CO_3_ and NaHCO_3_ pH 9.6) diluted 1/10 in PBS. The plates were incubated overnight at 4°C. Plates were washed with PBS-0.05% (v/v) Tween-20 (Sigma-Aldrich, UK) and blocked with PBS-0.05% (v/v) Tween-20 and 8% (w/v) skimmed milk powder (microbiology grade; Sigma-Aldrich, UK) for 1 h at 37°C. Plates were washed with PBS-0.05% (v/v) Tween20 and incubated with different concentrations of human CD50-HIS recombinant protein (purchased from Life Technologies, UK) for 1 h at 37°C. Bound protein was detected using a specific anti-CD50 mouse monoclonal antibody (MEM-171; Biorad, UK) and a secondary peroxidase-conjugated rabbit-anti-mouse monoclonal antibody (Abcam, Cambridge, UK). Peroxidase activity was detected with 3,3′,5,5′-Tetramethylbenzidine (TMB) liquid substrate (Sigma-Aldrich, UK) for 40 s and the reaction was stopped using 1M H_2_SO_4_. Absorbance was measured at 450 nm using a Synergy HT plate reader (BioTek, Vermont, USA).

### T-cell proliferation assay

Human PBMC were adjusted to a concentration of 1x10^6^ cells/ml in RPMI 1640 (Sigma Aldrich, UK) supplemented with 10% (v/v) heat-inactivated fetal calf serum (Gibco, UK), 100 U/ml penicillin, 100 μg/ml streptomycin and 292 μg/ml L-glutamine. (PSG) (Gibco, UK). Cells were cultured in 96-well round bottomed tissue culture plates (Nunc, Fisherbrand, UK) at 37°C in humidified air with 5% CO_2_. Protein samples were tested in triplicate and added at varying concentrations before incubation. Cells were cultured in medium only or with 1 μg/ml of Concanavalin A as negative and positive controls respectively. Proliferation of cells was determined using the incorporation of [^3^H]-thymidine, by pulsing with 1 μCi/well of [^3^H]-thymidine, after a 72 h incubation, and harvested after 18 h using a Tomtec Mach III M Harvester 96 (Hamden, USA) onto Wallac A filters (Perkin Elmer, MA, USA). A Meltilex A wax scintillant strip (Perkin Elmer, MA, USA) was melted onto the filter pad and the [^3^H]-thymidine incorporation into cellular DNA was determined by scintillation counting using a β-radiation counter (Wallac 1450 Microbeta PLUS, Perkin Elmer) and recorded as counts per minute (CPM).

### Rabbit necrotizing pneumonia rabbit model

The rabbit pneumonia model was performed as described previously with some modification [6]. Briefly, wild-type LAC and the *selx* mutants (Table 1) were cultured in Todd Hewitt broth for 16 h and washed once in Todd Hewitt broth. The bacteria were re-suspended in Todd Hewitt broth at 1×10^10^ CFU/ml for use in injections. New Zealand White rabbits (3–4 per group) were anesthetized with ketamine and xylazine. Their tracheas were exposed and a high (6x10^9^ CFU) or low (2×10^9^ CFU) dose of USA300 CA-MRSA strain LAC or mutant derivatives (Table 1) were administered intra-tracheally through catheters in 0.4 ml volumes. The animals were closed and monitored for 4 d for development of fatal necrotizing pneumonia.

### Murine skin abscess model

Female, inbred 8–12 week old female BALB/cOlaHsd mice aged between 8–12 weeks were obtained from ENVIGO (UK) and acclimatised for 1 to 2 weeks in the facility before being used in infection challenge studies. All mice were housed under specific pathogen-free conditions in individually ventilated cages (IVCs) at the Bioresearch Facility (BRF) of the Roslin Institute (University of Edinburgh, UK). 1 day prior to infection challenge, mice were anesthetized with isoflurane and the fur on the back of each animal was removed by using clippers. Overnight cultures of *S*. *aureus* were inoculated 1:100 into fresh BHI broth and cultured to mid-log phase (OD_600 =_ 0.6; approximately 2 h) with shaking at 37°C. Staphylococci were harvested by centrifugation, washed, and suspended in sterile PBS to obtain an inoculum of 1x10^8^ CFU/ml. Inocula were determined by CFU enumeration following serial dilution, plating on TSA plates, and overnight growth at 37°C. Prior to inoculation the animals were weighed and then every 24 h post infection. Mice were inoculated with 1x10^7^ CFU by subcutaneous injection to a depth of 5 mm, in the shaved area of skin on the back of each mouse. Infected animals were monitored for health status and lesion development over a period of 6 d using a standardized and Home Office project licence approved monitoring protocol. The size of each skin lesion was measured each day (one measurement across the longest dimension of the lesion). Bacterial tissue load was determined post-mortem, the skin lesion was excised, weighed and homogenised in 1 ml of sterile PBS using fast prep tubes containing lysis matrix D (MP Biomedicals, UK). Bacterial loads were determined by CFU enumeration following serial dilution, plating on TSA plates, and overnight growth at 37°C. CFU were normalised to tissue weight. For histopathological analysis lesions were excised and fixed in 10% neutral buffered formalin (NBF) (Leica microsystems, UK) for 24 h. Lesions were processed to parrifin blocks, sectioned and then stained with haematoxylin and eosin by the Veterinary Pathology Service Unit of the Royal (Dick) School of Veterinary Studies (University of Edinburgh).

### Statistical analysis

All statistical analysis was performed in Graphpad Prism 7. Grouped data was analysed to determine if a Gaussian distribution was true with the Shapiro-Wilk normality test. Parametric data was analysed using student t-test with Welches correction if required. Tests were unpaired and two-tailed and significant differences were considered when the p-value was <0.05. Protein dose curves were tested using two-way ANOVA analysis, multiple comparisons were performed using Sidak’s method. Animal survival curves were plotted using Kaplan-Meier method and significance was determined using log-rank (Mantel-Cox) analysis (p-value <0.05).

## Supporting information

S1 Fig Analysis of SElX-binding to different lymphocyte subpopulations. (i) Flow cytometery analysis of SElX binding human neutrophils, monocytes and lymphocytes indicating the relative binding of SElX to each cell type. SElX binding was determined using a FITC conjugate mouse anti-HIS-tag antibody. (ii) Two-colour flow cytometry was used to analyse SElX-binding to different lymphocyte subpopulations. T-lymphocytes (CD4^+^ and CD8^+^), and B-lymphocytes (CD19^+^) were concurrently stained with PE-conjugated antibodies directed against CD4, CD8, or CD19. SElX binding was determined using a FITC conjugate mouse anti-HIS-tag antibody.
(PDF)

S2 Fig Mutations in the predicted sialic acid-binding site of SElX have limited impact on protein structure and stability. Circular dichroism analysis was performed on SElX sialic acid-binding mutants to ensure that protein structures were not affected by the mutations (i). Thermal shift assays were performed to analyse protein stability (ii). The T_m_ of each mutant was determined by calculating the temperature at which the fluorescence peaked (excitation/emission/ 470/570 nm).
(PDF)

S3 Fig Phenotypic and genetic validation *of the LACΔselx mutant*, *LACΔselx rep and LAC selx EKQD-A strains*. (i) Growth curves of *S*. *aureus* USA300 mutants (grown at 37°C for 18h) in either TSB or BHI broth. (ii) SDS-PAGE analysis of concentrated supernatant and cell wall associated (CWA) protein fractions at both mid and post exponential growth phases in BHI broth (1. LAC, 2. LAC *selx* EKQD-A, 3. LACΔ*selx* rep, 4. LACΔ*selx*). (iii)) Western blot analysis of SElX expression in; (1) LAC, (2) LAC *selx* EKQD-A, (3) LACΔ*selx* rep and (4) LACΔ*selx*, with SElX-specific IgY. (iv) Whole genome analysis of each mutant strain was performed, Black ticks on coloured rings indicate the position of non-synonymous SNPs in wild-type and the three mutant strains relative to the USA300 FPR3757 (NCBI: NC_007793) chromosome sequence. SNPs that are not common to all four strains are labelled. Figure panel was generated using BRIG and custom scripts (PMID: 21824423)
(PDF)

S4 Fig SElX does not cause necrosis or apoptosis in human leukocytes. (i) LDH release assays were performed on human neutrophils to assess the necrotic potential of SElX compared to LukAB, employed as a positive control. Percentage lysis was determined relative to complete lysis of the neutrophils observed after addition of 0.2% (v/v) Trition-X to the cells. Results shown are the means of three different human donors, ± SD of the mean. (ii) Analysis of SElX-induced leukocyte apoptosis using DRAQ5 nuclear stain. Binding assays of SElX and human leukocytes were performed followed by addition of the nuclear stain DRAQ5 to determine apoptosis as determined by nuclear fragmentation. Quadrat plots show the binding of SElX along the X-axis and the Y-axis shows nuclear fragmentation indicated by higher DRAQ5 fluorescence.
(PDF)

S1 Table Neutrophil protein ligands of SElX identified by affinity precipitation analysis. (PDF)
