# Supplementary figures and images for: The Staphylococcus aureus superantigen SElX is a bifunctional toxin that inhibits neutrophil function
Source: PLoS Pathog. 2017 Sep 7;13(9):e1006461. doi: 10.1371/journal.ppat.1006461 (PMC5589267; doi:10.1371/journal.ppat.1006461)

**Figure S1**

**(i)**

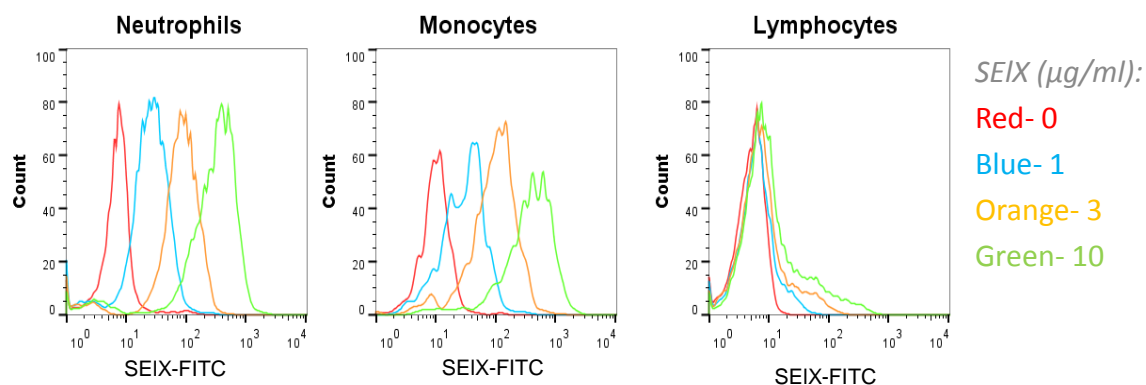

**(ii)**

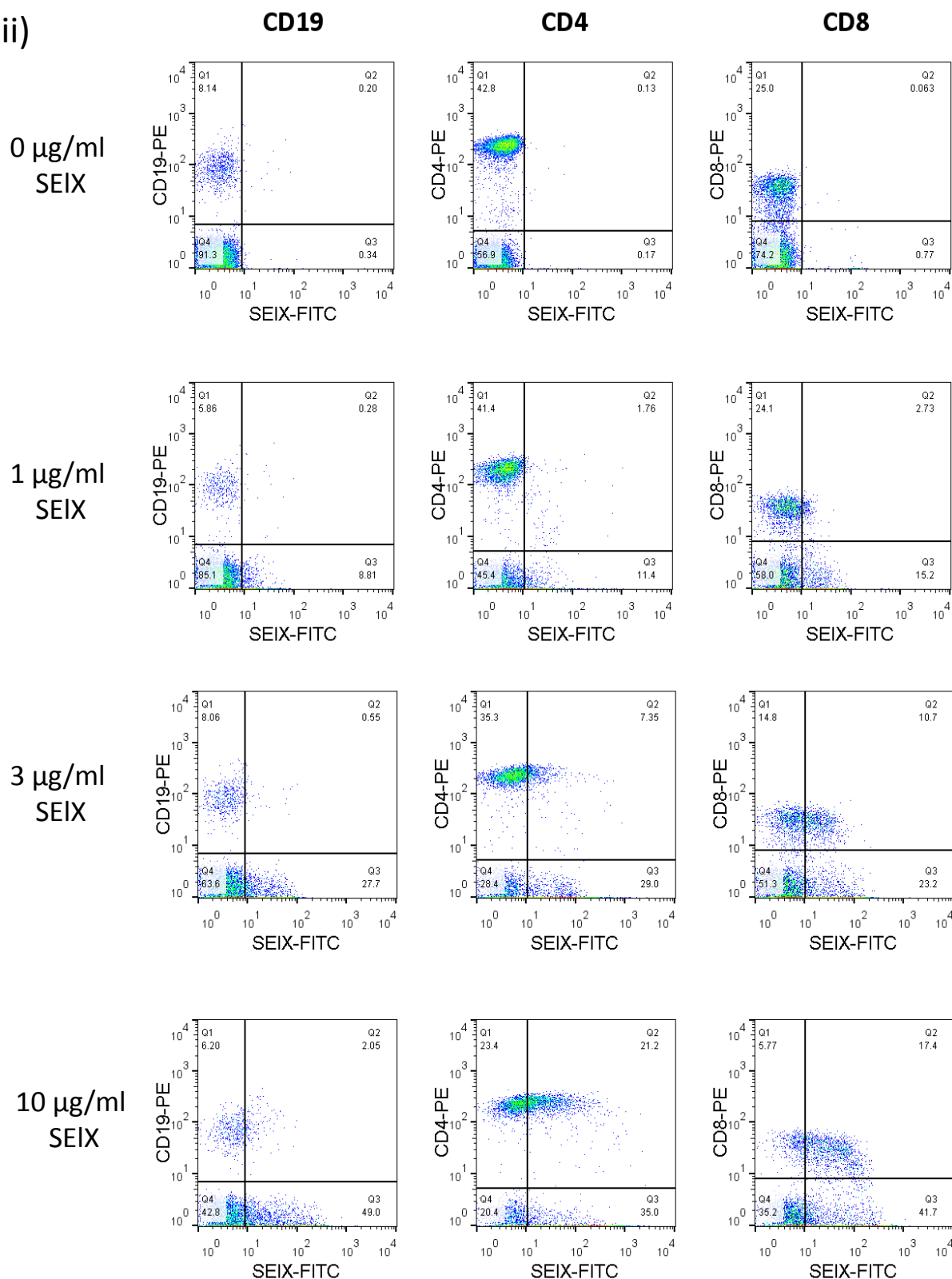

Supplement: S1 Fig — (i) Flow cytometery analysis of SElX binding human neutrophils, monocytes and lymphocytes indicating the relative binding of SElX to each cell type. SElX binding was determined using a FITC conjugate mouse anti-HIS-tag antibody. (ii) Two-colour flow cytometry was used to analyse SElX-binding to different lymphocyte subpopulations. T-lymphocytes (CD4+ and CD8+), and B-lymphocytes (CD19+) were concurrently stained with PE-conjugated antibodies directed against CD4, CD8, or CD19. SElX binding was determined using a FITC conjugate mouse anti-HIS-tag antibody. (PDF) [file ppat.1006461.s001.pdf]

**Figure S3**

**(i)**

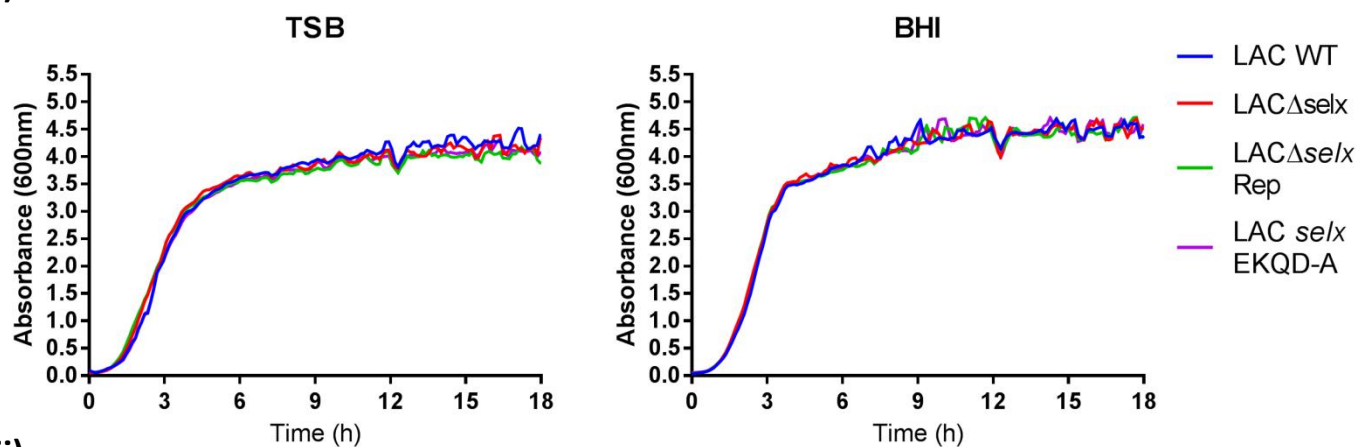

**(ii)**

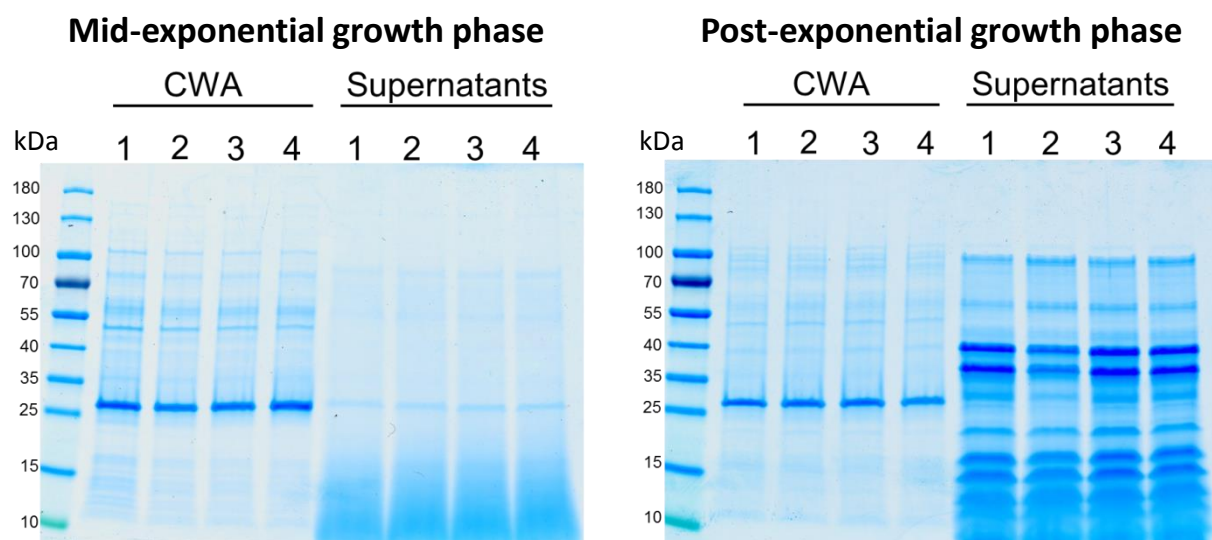

**(iii)**

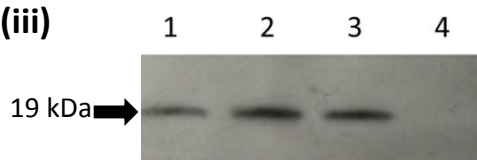

**(iv)**

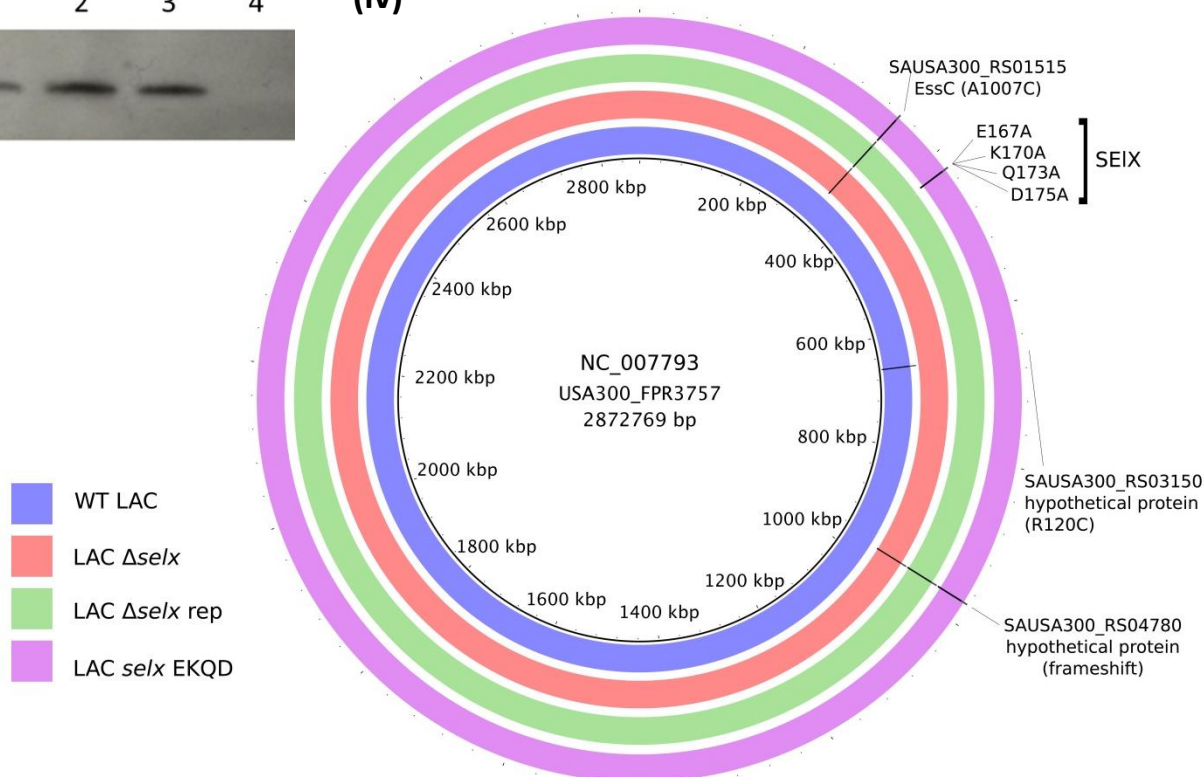

Supplement: S3 Fig — (i) Growth curves of S. aureus USA300 mutants (grown at 37°C for 18h) in either TSB or BHI broth. (ii) SDS-PAGE analysis of concentrated supernatant and cell wall associated (CWA) protein fractions at both mid and post exponential growth phases in BHI broth (1. LAC, 2. LAC selx EKQD-A, 3. LACΔselx rep, 4. LACΔselx). (iii)) Western blot analysis of SElX expression in; (1) LAC, (2) LAC selx EKQD-A, (3) LACΔselx rep and (4) LACΔselx, with SElX-specific IgY. (iv) Whole genome analysis of each mutant strain was performed, Black ticks on coloured rings indicate the position of non-synonymous SNPs in wild-type and the three mutant strains relative to the USA300 FPR3757 (NCBI: NC_007793) chromosome sequence. SNPs that are not common to all four strains are labelled. Figure panel was generated using BRIG and custom scripts (PMID: 21824423) (PDF) [file ppat.1006461.s003.pdf]

Figure S4

(i)

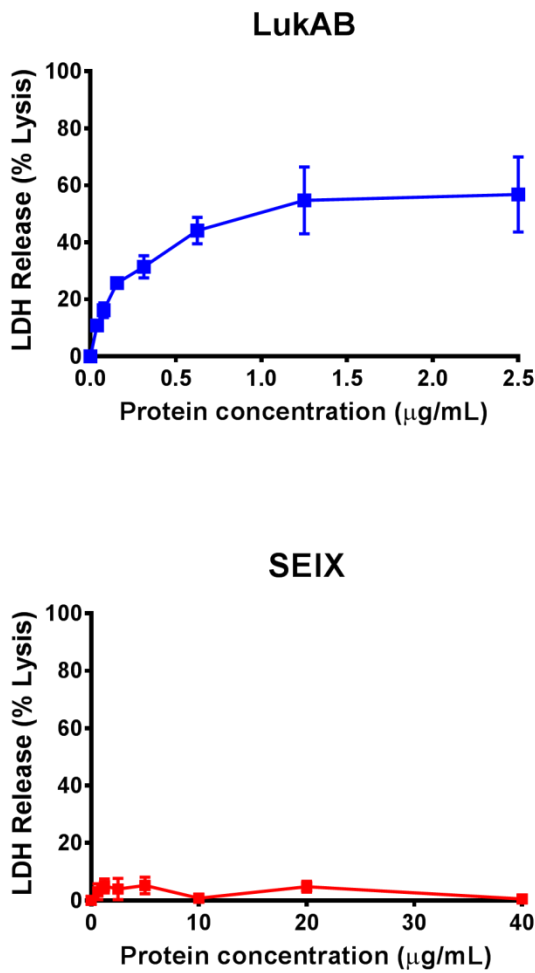

(ii)

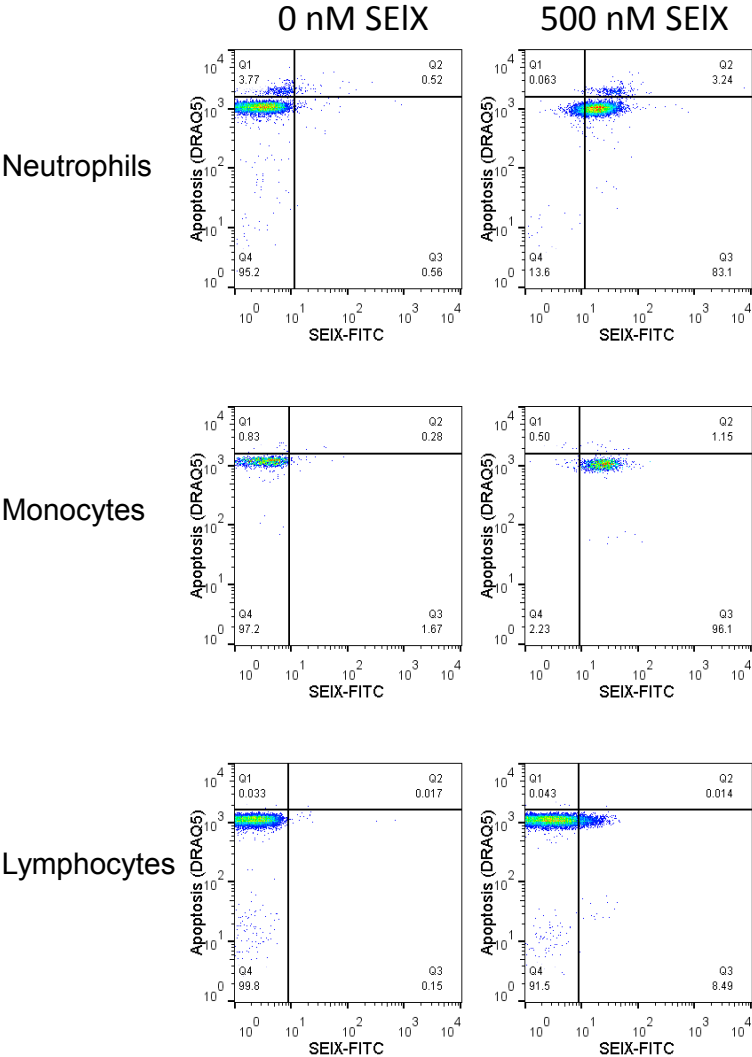

Supplement: S4 Fig — (i) LDH release assays were performed on human neutrophils to assess the necrotic potential of SElX compared to LukAB, employed as a positive control. Percentage lysis was determined relative to complete lysis of the neutrophils observed after addition of 0.2% (v/v) Trition-X to the cells. Results shown are the means of three different human donors, ± SD of the mean. (ii) Analysis of SElX-induced leukocyte apoptosis using DRAQ5 nuclear stain. Binding assays of SElX and human leukocytes were performed followed by addition of the nuclear stain DRAQ5 to determine apoptosis as determined by nuclear fragmentation. Quadrat plots show the binding of SElX along the X-axis and the Y-axis shows nuclear fragmentation indicated by higher DRAQ5 fluorescence. (PDF) [file ppat.1006461.s004.pdf]
