# Supplementary material for: The Staphylococcus aureus superantigen SElX is a bifunctional toxin that inhibits neutrophil function
Source: PLoS Pathog. 2017 Sep 7;13(9):e1006461. doi: 10.1371/journal.ppat.1006461 (PMC5589267; doi:10.1371/journal.ppat.1006461)
Supplement: S2 Fig — Circular dichroism analysis was performed on SElX sialic acid-binding mutants to ensure that protein structures were not affected by the mutations (i). Thermal shift assays were performed to analyse protein stability (ii). The Tm of each mutant was determined by calculating the temperature at which the fluorescence peaked (excitation/emission/ 470/570 nm). (PDF) [file ppat.1006461.s002.pdf]

Figure S2

(i)

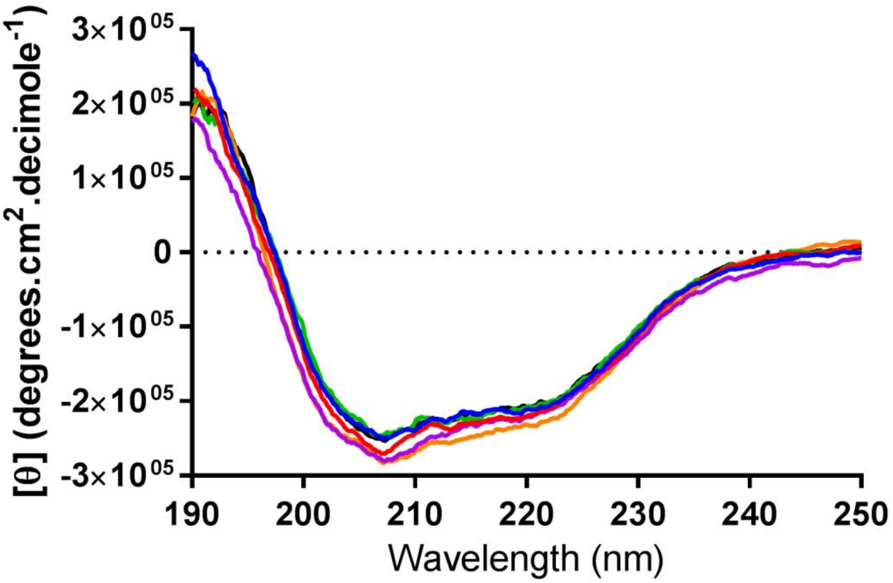

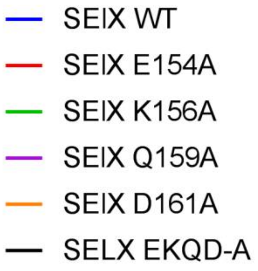

(ii)

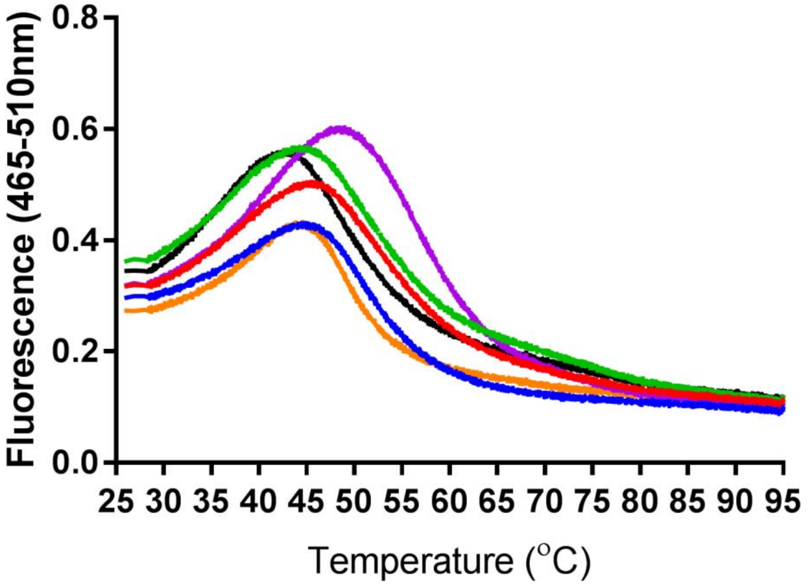

**SElX mutant**	**T_m (°C)**
WT	44.88
E154A	45.62
K156A	43.70
Q159A	48.80
D161A	43.89
EKQD-A	42.63
