# Supplementary material for: The Staphylococcus aureus superantigen SElX is a bifunctional toxin that inhibits neutrophil function
Source: PLoS Pathog. 2017 Sep 7;13(9):e1006461. doi: 10.1371/journal.ppat.1006461 (PMC5589267; doi:10.1371/journal.ppat.1006461)
Supplement: S1 Table — (PDF) [file ppat.1006461.s005.pdf]

Table S1: Neutrophil protein ligands of SElX identified by affinity precipitation analysis

Protein	Size (kDa)	Accession[a]
CD45	130.8	P08575-2
MGAM (maltase-glucoamylase)	311	E7ER45
CD31	109.5	P16284-3
CD13	80.2	P15144
CD148	145.9	Q12913
CR1 (CD35)	200	P17927
CD16b	38	O75015
p22-PHOX	65.3	P04839
CD50 (ICAM-3)	59.5	P32942
Integrin α-L (CD11a)	119.1	P20701-3
P-selectin	50	P16109
L-selectin	74	P16109
VNN2	60	O95498
C1q receptor (CD93)	68.6	Q9NPY3
ADP-ribosyl cyclase (CD38)	34.3	P28907
Nicastrin	78.4	Q92542
Alkaline phosphatase	57.3	P05186

[a] Uniprot accession numbers
